# Retinitis Pigmentosa‐Associated Gene *TRIM49* Regulates ULK1‐Mediated Autophagy and Photoreceptor Phagocytosis by the Retinal Pigment Epithelium

**DOI:** 10.1002/advs.202512305

**Published:** 2025-09-16

**Authors:** Zhen Yi, Chaojuan Wen, Han Du, Yingwei Wang, Shuowei Chen, Jie Liu, Shuhan Zhang, Junwen Wang, Xueshan Xiao, Shiqiang Li, Xiaoyun Jia, Yi Jiang, Jiamin Ouyang, Panfeng Wang, James Fielding Hejtmancik, Wenmin Sun, Huangxuan Shen, Qingjiong Zhang

**Affiliations:** ^1^ State Key Laboratory of Ophthalmology Zhongshan Ophthalmic Center Guangdong Provincial Key Laboratory of Ophthalmology and Visual Science Sun Yat‐sen University Guangzhou 510060 China; ^2^ Molecular Ophthalmic Genetics Section Ophthalmic Genetics and Visual Function Branch National Eye Institute Rockville MD 20852 USA; ^3^ Ophthalmic Molecular Genetics State Key Laboratory of Ophthalmology Zhongshan Ophthalmic Center Sun Yat‐sen University Guangzhou 510060 China; ^4^ Biobank of Eye State Key Laboratory of Ophthalmology Zhongshan Ophthalmic Center Sun Yat‐sen University Guangzhou 510060 China

**Keywords:** autophagy, neurodegeneration, photoreceptor phagocytosis, retinal pigment epithelium, TRIM49

## Abstract

Autophagy is pivotal for cellular homeostasis and photoreceptor outer segment (POS) phagocytosis by the retinal pigment epithelium (RPE) in retinal degenerative diseases, such as age‐related macular degeneration and retinitis pigmentosa (RP). Yet, the mechanism of autophagy in the RPE remains largely unknown. RP is an inherited retinal degenerative condition, whose candidate genes provide avenues for dissecting novel autophagy factors. Here, whole exome sequencing of RP patients identified biallelic variants in *TRIM49*, a primate‐specific gene involved in autophagy, as a novel cause of RP. Among human tissues TRIM49 is highly expressed in the RPE. In human RPE cells, deficiency of *TRIM49* significantly disturbs cellular homeostasis and impairs the POS phagocytosis. Importantly, suppressed basal autophagy flux is present in *TRIM49*‐depleted RPE cells, whereas enhanced autophagy flux is present in RPE cells overexpressing *TRIM49*. Variations of TRIM49 after treatment with multiple autophagy modulators indicate that *TRIM49* is involved in the initiation of autophagy. TRIM49 interacts with the key regulator of autophagy initiation ULK1. Deficiency of *TRIM49* down‐regulates and overexpression of *TRIM49* up‐regulates ULK1 expression. Altogether, these findings identify *TRIM49* as a critical regulator of ULK1‐mediated autophagy and POS phagocytosis by the RPE, suggesting that RPE autophagy is a potential therapeutic target for retinal degenerative diseases.

## Graphical abstract

Based on next‐generation sequencing data of retinitis pigmentosa (RP) patients and controls, this study identifies *TRIM49* as a novel gene for autosomal recessive RP. *TRIM49* is specifically expressed in the retinal pigment epithelium (RPE) of human retina. Cellular and molecular biological assays demonstrate that *TRIM49* deficiency suppresses ULK1‐mediated autophagy, reduces CD36 levels and impairs photoreceptor phagocytosis of RPE, mimicking RP patients. Furthermore, the transcription factor OTX2 regulates *TRIM49* expression.

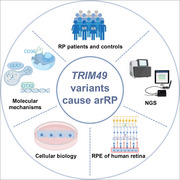

## 1 Introduction

Autophagy is a lysosome‐mediated degradation process involving the engulfment of cytoplasmic material within double‐membrane autophagosomes, maturation of the autophagosomes, and eventual fusion with lysosomes for degradation.^[^ ^1^ ^]^ Autophagy maintains cellular homeostasis and responses to environmental stress by removing toxic aggregates, damaged organelles, and misfolded proteins.^[^ ^2^, ^3^ ^]^ It generally acts as a cytoprotective mechanism for neuronal survival.^[^ ^4^ ^]^ The suppression of autophagy has been implicated in the accumulation of cytoplasmic inclusions and SQSTM1 aggregates in neurons and in massive neuronal death, i.e., neurodegeneration.^[^ ^5^, ^6^ ^]^

The vertebrate retina, as many other central nervous system structures, consists of extensively diverse neuronal types that form a highly ordered neuronal network.^[^ ^7^ ^]^ Retinitis pigmentosa (RP) is a collection of inherited retinal dystrophies (IRD) characterized by progressive degeneration of photoreceptors and the retinal pigment epithelium (RPE), of which the causative genes and variants remain unknown in about half of the cases.^[^ ^8^, ^9^, ^10^, ^11^, ^12^ ^]^ Monogenic variants that cause RP are predominantly in genes expressed in photoreceptors or RPE.^[^ ^13^ ^]^

The RPE is a monolayer of epithelial cells that lie close to the photoreceptor outer segment (POS) of the neural retina, and carries out many activities to support the function and survival of photoreceptor neurons and therefore vision.^[^ ^14^, ^15^ ^]^ As the most active phagocytic cells in the human body, daily POS phagocytosis is a key function of the RPE.^[^ ^16^ ^]^ Photoreceptor outer segments are subjected to sustained light exposure that generates high levels of oxidative stress, and require continuous membrane renewal to ensure lifelong functioning.^[^ ^17^, ^18^ ^]^ The RPE promptly phagocytizes the enormous loads of fragments shed by the adjacent POS and recycles their components.^[^ ^15^, ^19^ ^]^ If the cell health and POS phagocytosis by the RPE are impaired, the accumulation of debris in the subretinal space leads to photoreceptor degeneration and thus loss of vision.^[^ ^20^, ^21^ ^]^

Autophagy plays a central role in maintaining RPE health and POS phagocytosis, digesting and recycling intracellular and POS components in lysosomes in response to light and stress conditions.^[^ ^15^, ^22^, ^23^ ^]^ Autophagy is upregulated in the RPE in response to cell stress, and impaired autophagy is a feature of a plethora of photoreceptor degenerations. Knock out of *RB1CC1*, a component of the ULK complex required to initiate autophagy, results in the degeneration of the RPE and a secondary loss of photoreceptors.^[^ ^24^ ^]^ Mice deficient in *Epg5*, a gene required for autophagosome maturation, exhibit SQSTM1 aggregates in the RPE and features of RP.^[^ ^25^ ^]^ The autophagy proteins ATG5 and BECN1/Beclin 1 are involved in LC3‐associated phagocytosis, which has recently emerged as a major mechanism for the degradation of shed POS in the RPE.^[^ ^26^ ^]^ Moreover, deficiency of *CERKL*, a RP causative gene, results in reduced basal autophagy in photoreceptors and RPE cells prior to the onset of photoreceptor death.^[^ ^27^ ^]^ Tripartite motif (TRIM) family proteins are a group of innate immune effectors and function as autophagy receptors and regulators of autophagosome formation.^[^ ^28^, ^29^, ^30^, ^31^ ^]^ To our knowledge, among TRIM family members, only *TRIM32* is known to be associated with IRD thus far. Patients harboring homozygous *TRIM32* missense variants displayed Bardet–Biedl syndrome (a form of syndromic RP), including obesity, RP, hypogonadism, and cognitive impairment.^[^ ^32^ ^]^ Interestingly, *TRIM49*, a member of the TRIM family, shows retina‐specific mRNA expression in humans (GeneCards‐ SAGE, https://www.genecards.org/) but has not yet been functionally characterized.

In this study, we report the identification of biallelic *TRIM49* variants in non‐syndromic RP patients from two unrelated families. Further analyses of its expression profile reveals that the TRIM49 protein is specifically found in the RPE of the human retina. Because *TRIM49* has no orthologs in rodents or other common laboratory animals^[^ ^33^ ^]^ and is specifically expressed in human RPE cells, further functional studies of *TRIM49* were conducted in primary human RPE cells and the cell lines hTERT RPE‐1 and ARPE‐19. Studies of *TRIM49* knockdown or overexpression in human RPE cells demonstrate that *TRIM49* is essential for supporting cellular homeostasis, POS phagocytosis, and autophagic flux by maintaining expression of ULK1 in the human RPE. *TRIM49* deficiency defines a novel form of RP associated with defects in autophagy and POS phagocytosis by the RPE, making *TRIM49* is one of the rare autophagy genes found to be mutated in non‐syndromic RP.

## 2 Results

### 2.1 Whole Exome Sequencing of RP Patients Identifies Homozygous Rare Variants in *TRIM49*

**Figure 1:**
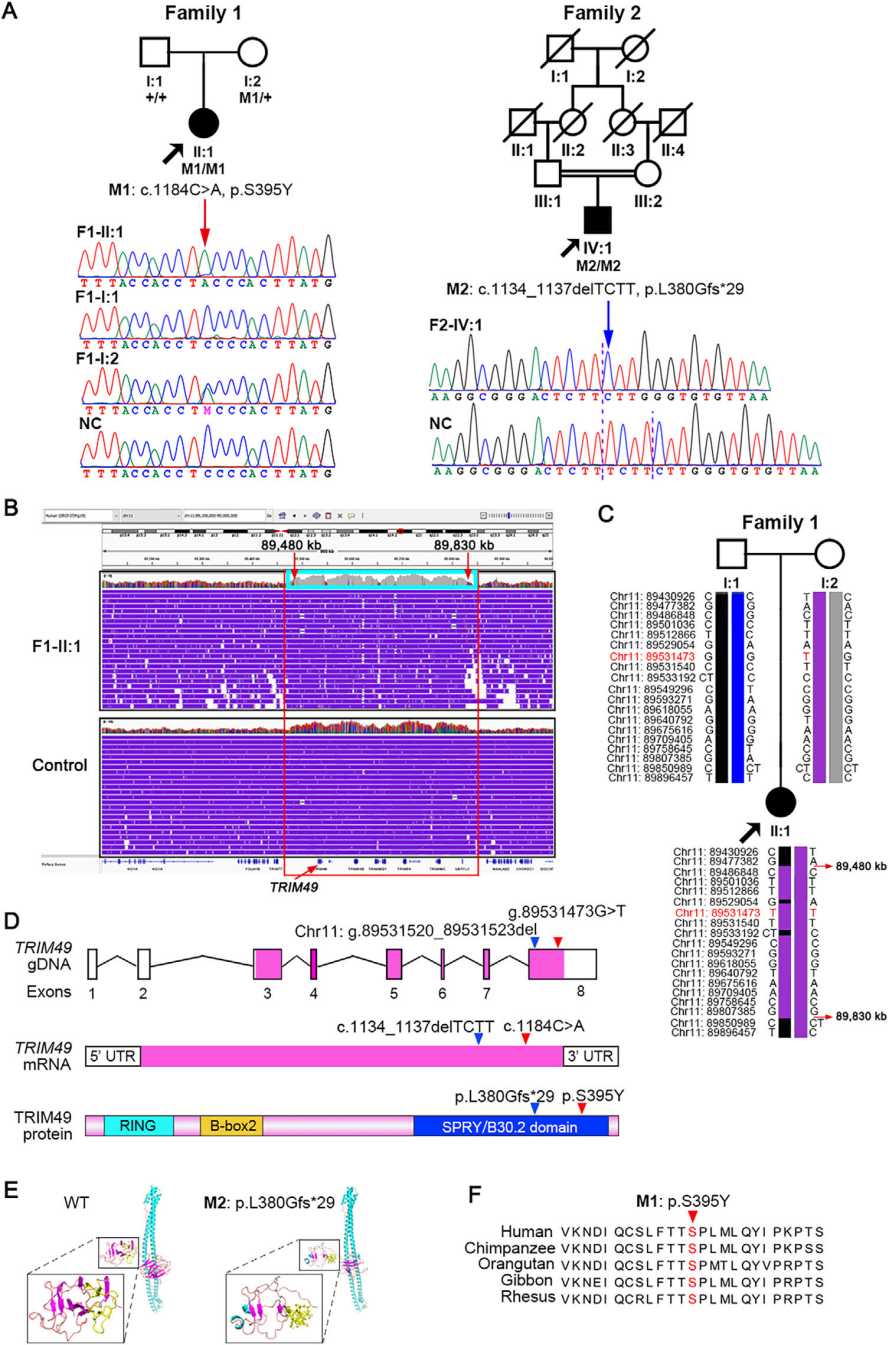
*TRIM49* homozygous variants identified in two unrelated families with RP. A) The pedigrees, variant segregation data and sequence chromatograms in two families. The family numbers are indicated above the corresponding pedigree. The genotypes for available family members are shown below each individual. Blackened symbols represent affected individuals. Mx, mutant alleles; +, wild‐type allele. The double horizontal lines represent consanguineous marriage. B) Integrative Genomics Viewer (IGV) of long‐read genome sequencing data from F1‐II:1 (upper panel) and a control individual (lower panel). The grey alignments (inside the blue box) in F1‐II:1 represent a homozygous region of ≈350 kb (from ≈89480 to ≈89830 kb in chromosome 11, covering *TRIM49* gene). The read depth of the grey alignments is comparative to that of the control individual, inconsistent with a large deletion and suggesting that there may be maternal UPD in F1‐II:1. C) Local SNP haplotypes for the c.1184C>A (chr11:89531473 G>T) variant based on trio whole genome sequencing in Family 1. The homozygous region detected by long‐read genome sequencing (B) is confirmed by local SNP haplotypes in F1‐II:1. Local SNP haplotypes of the father (F1‐I:1) show heterozygosity throughout the homozygous region in F1‐II:1, which is also inconsistent with a large deletion and suggesting that there may be maternal UPD in F1‐II:1. In the ≈350 kb homozygous region of F1‐II:1, some SNPs may be masked by pseudogenes, such as chr11: 89529054 and chr11: 89533192. D) Upper panel: Schematic representation of the genomic structure of *TRIM49* (GenBank: NM_020358.2) showing the location of M1 and M2 identified in the two families. Numbers below the diagram indicate the corresponding exon number. Middle panel: Schematic representation of the messenger RNA of *TRIM49*. Lower panel: Schematic showing the domains of the TRIM49 protein. Both affected amino acids of the two variants are located in the B30.2 domain. The RING finger spans amino acids 9–67, the B‐box2 spans amino acids 87–139, and the B30.2 domain spans amino acids 285–448. UTR, untranslated region. E) Structure modelling of wild‐type and mutant TRIM49 proteins. Model of TRIM49 mutant product due to the p.L380Gfs*29 variant (right column) exhibits significant predicted structural perturbation in region of C‐terminal binding interface when compared to model of wild‐type protein (left column). F) Sequence alignment shows conservation of the affected amino acid (serine) in TRIM49 across various primate organisms. *TRIM49* is a primate‐specific gene with no homologous genes in rodents or other common laboratory animals. Arrowheads, the variant site.

Two rare homozygous pathogenic variants in *TRIM49* for non‐syndromic RP were identified using a comparative analysis of whole exome sequencing (WES) data from two unrelated probands with RP and 7283 in‐house controls. The 7283 unrelated control individuals are with ocular conditions other than RP, including 492 unaffected individuals (i.e., normal controls), 1561 probands with high myopia, 1231 probands with glaucoma, and 3999 probands with other genetic eye diseases. The two variants include a homozygous c.1184C>A (p.S395Y) variant in the proband from Family 1 (F1‐II:1) and a homozygous c.1134_1137delTCTT (p.L380Gfs*29) variant in the proband from Family 2 (F2‐IV:1), based on transcript NM_020358.2 (**Figure** **1**A). Both variants were validated with Sanger sequencing in the two probands (Figure 1A). Potentially pathogenic variants in genes known to cause IRD were not identified in either of the two probands, while several single heterozygous variants were detected in three genes responsible for autosomal recessive IRD (Table S1, Supporting Information). Structural variations or copy number variations involving these genes were not detected by WES in both probands while deep intronic variants of these genes have not been reported before. Sanger sequencing revealed that the variant c.1184C>A (p.S395Y) was present in the proband's mother in a heterozygous state but not in the father (Figure 1A). Paternity testing confirmed that the presumed father F1‐I:1 was the biological father of F1‐II:1 (Figure S1A, Supporting Information). Ultra‐high‐depth next‐generation sequencing (20000×, 682818 reads) revealed no mosaicism of the variant in DNA from F1‐I:1′s semen (Figure S1B, Supporting Information). Long‐read genome sequencing revealed a homozygous region of ≈350 kb in F1‐II:1 (Figure 1B). The origin of the other c.1184C>A (p.S395Y) variant in F1‐II:1 may be maternal uniparental disomy (UPD) or a large deletion mimicking homozygous variants. In the long‐read genome sequencing data, the read depth of the homozygous region in F1‐II:1 is similar to that of control individuals (Figure 1B), inconsistent with a large deletion and suggesting that there may be maternal UPD in F1‐II:1. Local SNP haplotypes show heterozygosity in the father (F1‐I:1) throughout the homozygous region in F1‐II:1 (Figure 1C), which is also inconsistent with a large deletion. In the ≈350 kb homozygous region of F1‐II:1, some SNPs may be masked by pseudogenes, such as chr11: 89529054 and chr11: 89533192 (Figure 1C). The p.S395Y variant is very rare, with an overall allele frequency of 0.0000041 in gnomAD and putative homozygote frequency of 1.68 × 10^−11^. The p.L380Gfs*29 variant is also rare, with an overall allele frequency of 0.0000082 in gnomAD. Moreover, biallelic potentially pathogenic variants in *TRIM49* were not present in 7283 in‐house controls, and no submissions of clinically relevant variants in *TRIM49* were found in Gene Matcher (https://genematcher.org/). Biallelic rare variants of a gene in two or more unrelated families with the same disease are extremely rare and potentially pathogenic.

Both variants are located in the C‐terminal SPRY (B30.2) domain of *TRIM49* (Figure 1D), which is known to contain a canonical binding interface that acts as the platform for the assembly of autophagy regulator complexes.^[^ ^29^, ^34^ ^]^ The p.L380Gfs*29 variant is a frameshift deletion variant predicted to significantly disrupt the spatial structure of the C‐terminal binding site (Figure 1E). In addition, the p.S395Y variant is a missense variant predicted to be disease‐causing using various pathogenicity prediction tools including PolyPhen‐2, PROVEAN, and SIFT (Table S2, Supporting Information). Phylogenic analysis revealed that *TRIM49* is a primate‐specific gene with no homologous genes in rodents or other common laboratory animals.^[^ ^33^ ^]^ In addition, serine (S) at amino acid position 395 in *TRIM49* is conserved among human and non‐human primate organisms (Figure 1F). These in‐silico bioinformatic findings suggest that the two homozygous *TRIM49* variants (p.L380Gfs*29 and p.S395Y) are potentially pathogenic for RP in these two families.

### 2.2 Clinical Features of the Two Unrelated RP Probands

**Figure 2:**
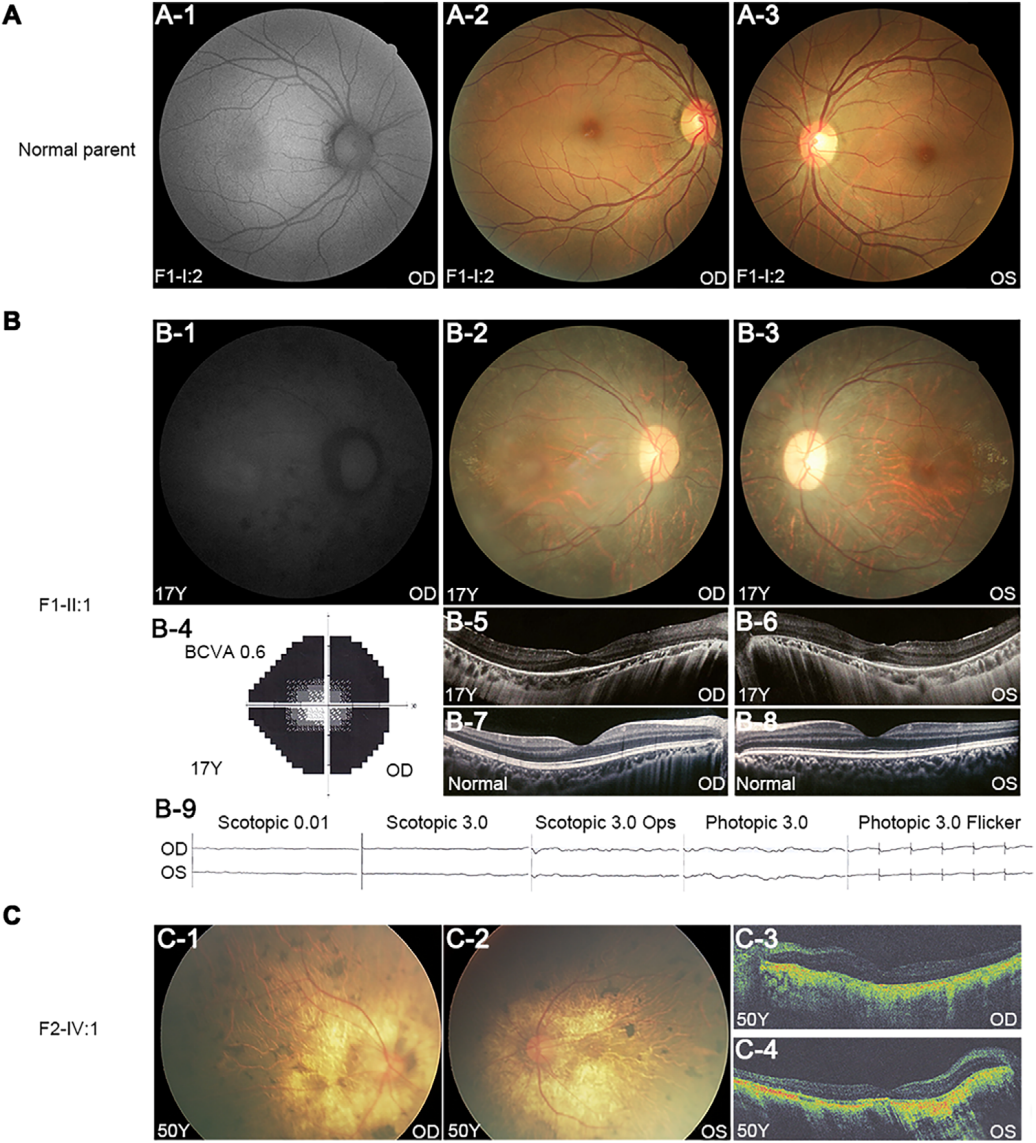
Clinical features of RP patients with homozygous *TRIM49* variants. A) FAF (A‐1) and color fundus photography (A‐2 and A‐3) from unaffected individual F1‐I:2 are provided as a normal control. B) FAF, color fundus photography, visual field, OCT, and full‐field ERG from the proband F1‐II:1 at age 17 years. Fundus photography of posterior pole reveal a pale optic disc, attenuated retinal blood vessels, tapetoretinal degeneration, and atrophy RPE (B‐2 and B‐3). FAF imaging showed marked weakness of autofluorescence (B‐1). Though with visual acuity of 0.6, significant constriction of the visual field could be observed (B‐4). Diffuse atrophy of the outer retinal layer with evident decline of the outer nuclear layer thickness and loss of the ellipsoid zone were found on OCT examination (B‐5 and B‐6). B‐7 and B‐8 are normal OCT images. Cone and rod responses are extinguished (B‐9). C) Color fundus photography and OCT from the proband F2‐IV:1 at age 50 years. The image of the posterior pole showed attenuated retinal arteries, apparent pigmentation in the mid‐peripheral retina, and a wide range of RPE atrophy (C‐1 and C‐2). Markedly thinned outer retina were seen on OCT scans (C‐3 and C‐4). BCVA, best‐corrected visual acuity; OD, right eye; OS, left eye; Y, years.

**Figure 3:**
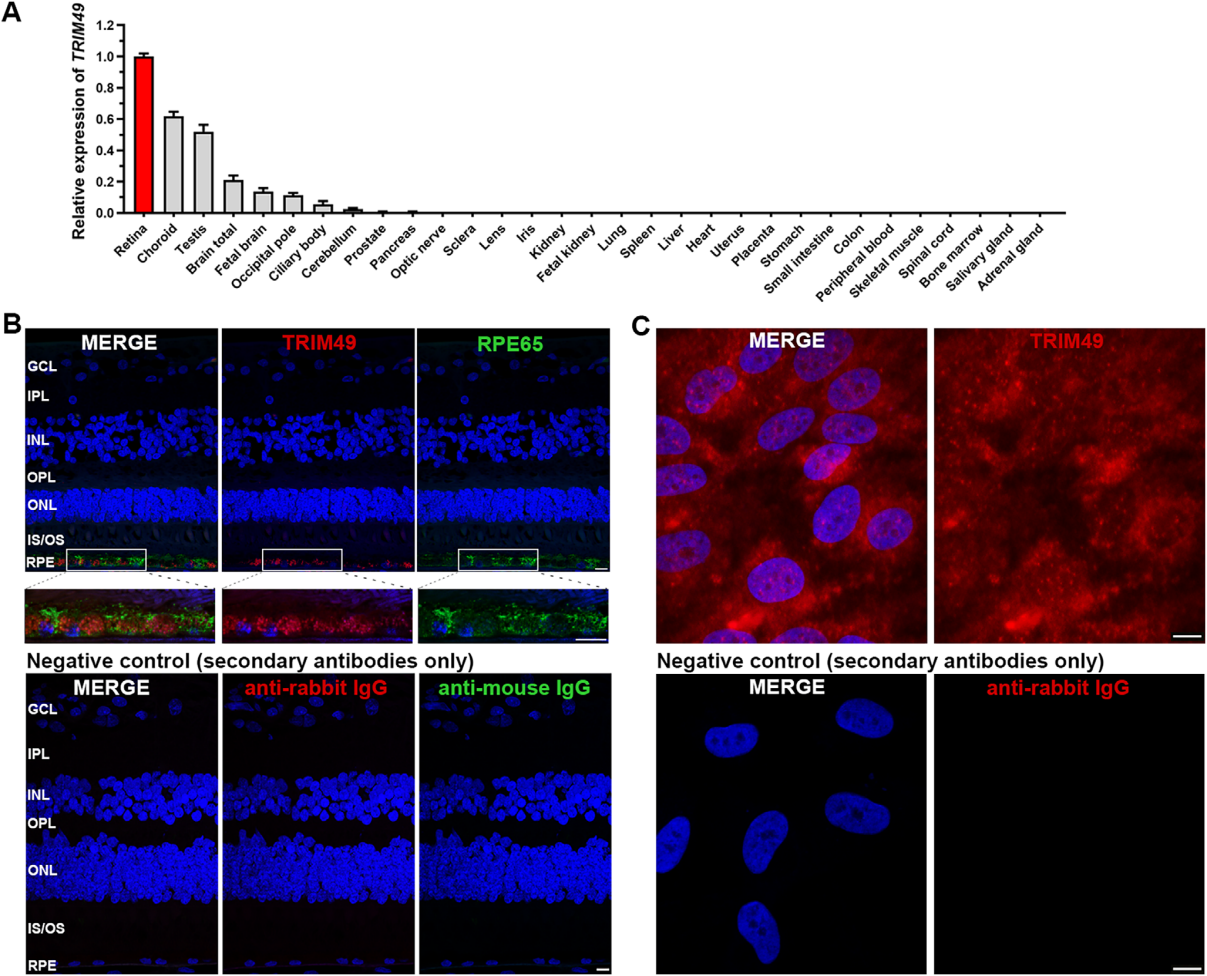
Expression profile of *TRIM49* in human tissues. A) *TRIM49* mRNA expression in adult and fetal human tissues relative to the retina. *TRIM49* had the highest transcription in retina. Tissues except fetal brain and fetal kidney were from adult human. *ACTB* was used as an internal control. Error bars represent the standard deviation of the relative quantities. B) Immunostaining of TRIM49 (red) and RPE65 (green) in human retina, revealing exclusive labelling for TRIM49 (red) in the RPE layer. C) Immunostaining of TRIM49 (red) in isolated human RPE cells for subcellular localization analyses, revealing that it is predominantly located in the cytoplasm and less in the nucleus of RPE cells. GCL, ganglion cell layer; IPL, inner plexiform layer; INL, inner nuclear layer; OPL, outer plexiform layer; ONL, outer nuclear layer; IS, inner segments of photoreceptors; OS, outer segments of photoreceptors; RPE, retinal pigment epithelium layer. The scale bar represents 10 µm.

**Figure 4:**
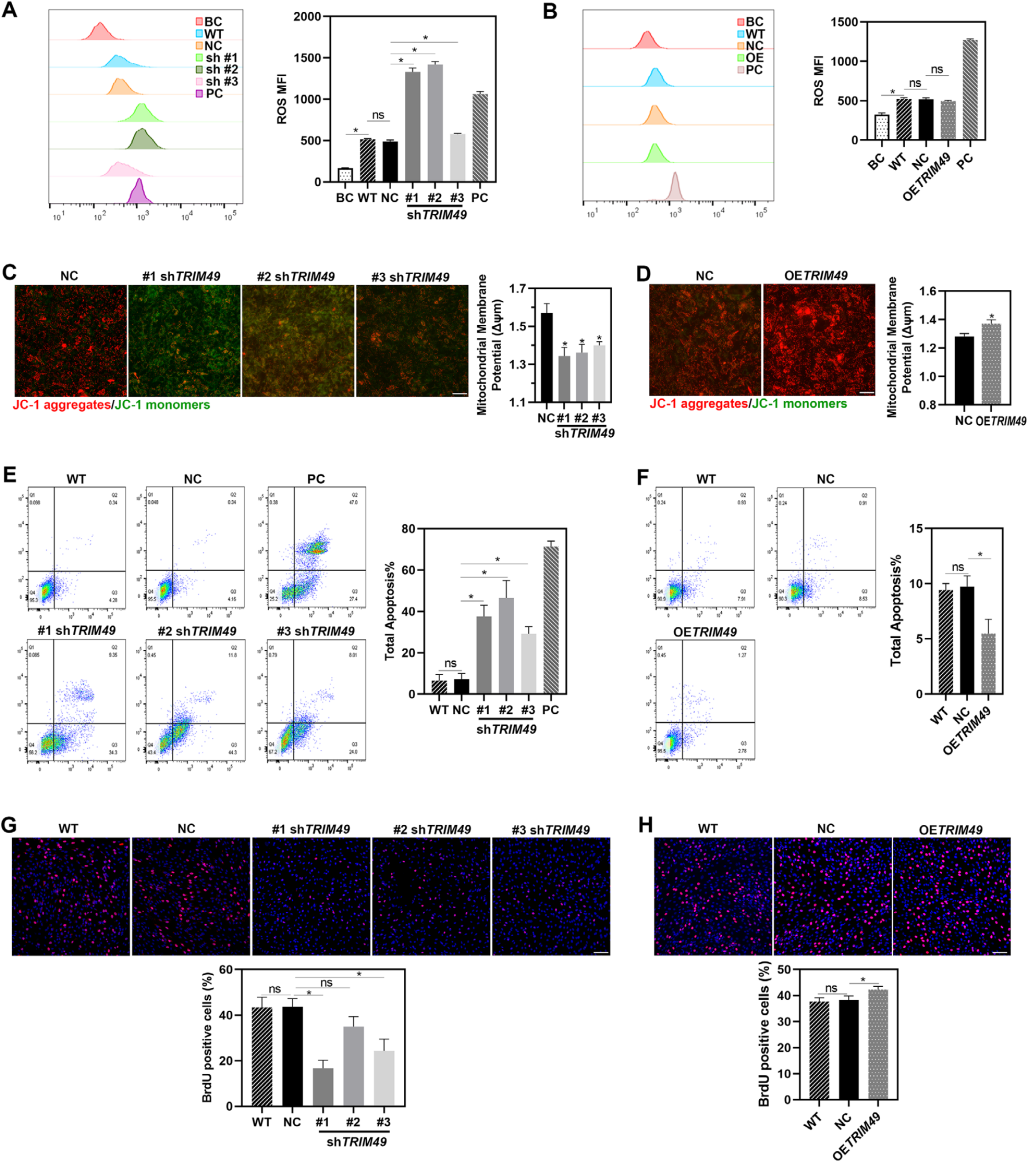
*TRIM49* is essential for the cell health of human RPE. A,B) Representative reactive‐oxygen species (ROS) MFI analyzed by flow cytometry. Elevated levels of ROS were observed in TRIM49‐depleted RPE cells (A). No significant difference was observed between ROS of normal control (NC) and TRIM49 overexpression RPE cells (B). The blank control (BC) is the negative staining control. Wild‐type (WT) RPE cells without any treatments are used as negative controls. NaIO3‐treated human RPE cells were used as positive controls (PC). C,D) Representative images of stained cells by JC‐1. JC‐1 aggregates are stained with red, and JC‐1 monomers are stained with green. A lower mitochondrial membrane potential (Δψm) were observed in TRIM49‐depleted RPE cells (C). A higher Δψm were observed in TRIM49 overexpression RPE cells (D). The scale bar represents 50 µm. E,F) Representative flow cytometric analyses of apoptosis rate. The cell apoptosis rate was elevated in TRIM49‐depleted RPE cells (E). The cell apoptosis rate was reduced in TRIM49 overexpression RPE cells (F). Wild‐type (WT) RPE cells without any treatments are used as negative controls. NaIO3‐treated human RPE cells were used as positive controls (PC). (G‐H) Inhibited cell proliferation was observed in TRIM49‐depleted RPE cells G) while enhanced cell proliferation was observed in TRIM49 overexpression RPE cells H). Wild‐type (WT) RPE cells without any treatments are used as negative controls. *P < 0.05 ns: no significance; OE: overexpression. *N* = 3 for each group. Error bars indicate standard deviation.

**Figure 5:**
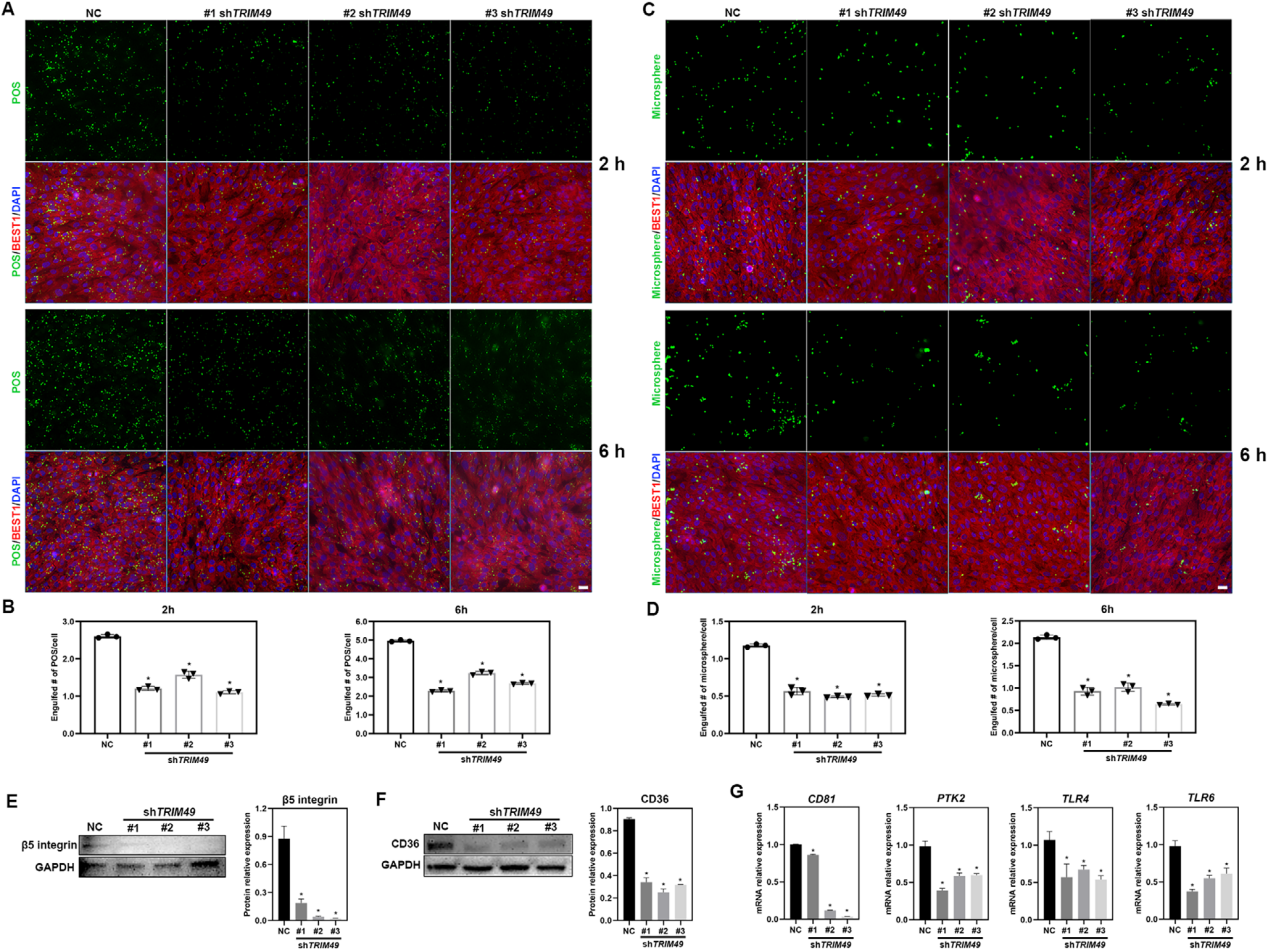
*TRIM49* deficiency impairs the phagocytic capacity of human RPE and downregulates phagocytic receptors expression. A) The hTERT RPE‐1 cells were challenged with 10 FITC‐labeled porcine photoreceptor outer segments (POS) per RPE cell for 2 and 6 h. POS (green), BEST1 (red), DAPI (blue). B) Quantification of engulfed POS in normal control and TRIM49‐depleted RPE cells after 2 and 6 h of treatment. C) The hTERT RPE‐1 cells were challenged with 10 microspheres per RPE cell for 2 and 6 h. Microsphere (green), BEST1 (red), DAPI (blue). D) Quantification of engulfed microspheres/cell by normal control and TRIM49‐depleted RPE cells after 2 and 6 h of treatment. E) Immunoblotting and quantification of β5 integrin expression in NC and TRIM49‐depleted RPE cells. F) Immunoblotting and quantification of CD36 expression in NC and TRIM49‐depleted RPE cells. G) The mRNA expression of upstream or downstream molecules of β5 integrin (CD81 and PTK2/FAK) and CD36 (TLR4 and TLR6) in NC and TRIM49‐depleted RPE cells. The expression of CD81, PTK2/FAK, TLR4, and TLR6 were noticeably decreased in TRIM49‐depleted hTERT RPE‐1 cells. *P < 0.05. Error bars indicate standard deviation. *N* = 3 for each group. The scale bar represents 25 µm.

**Figure 6:**
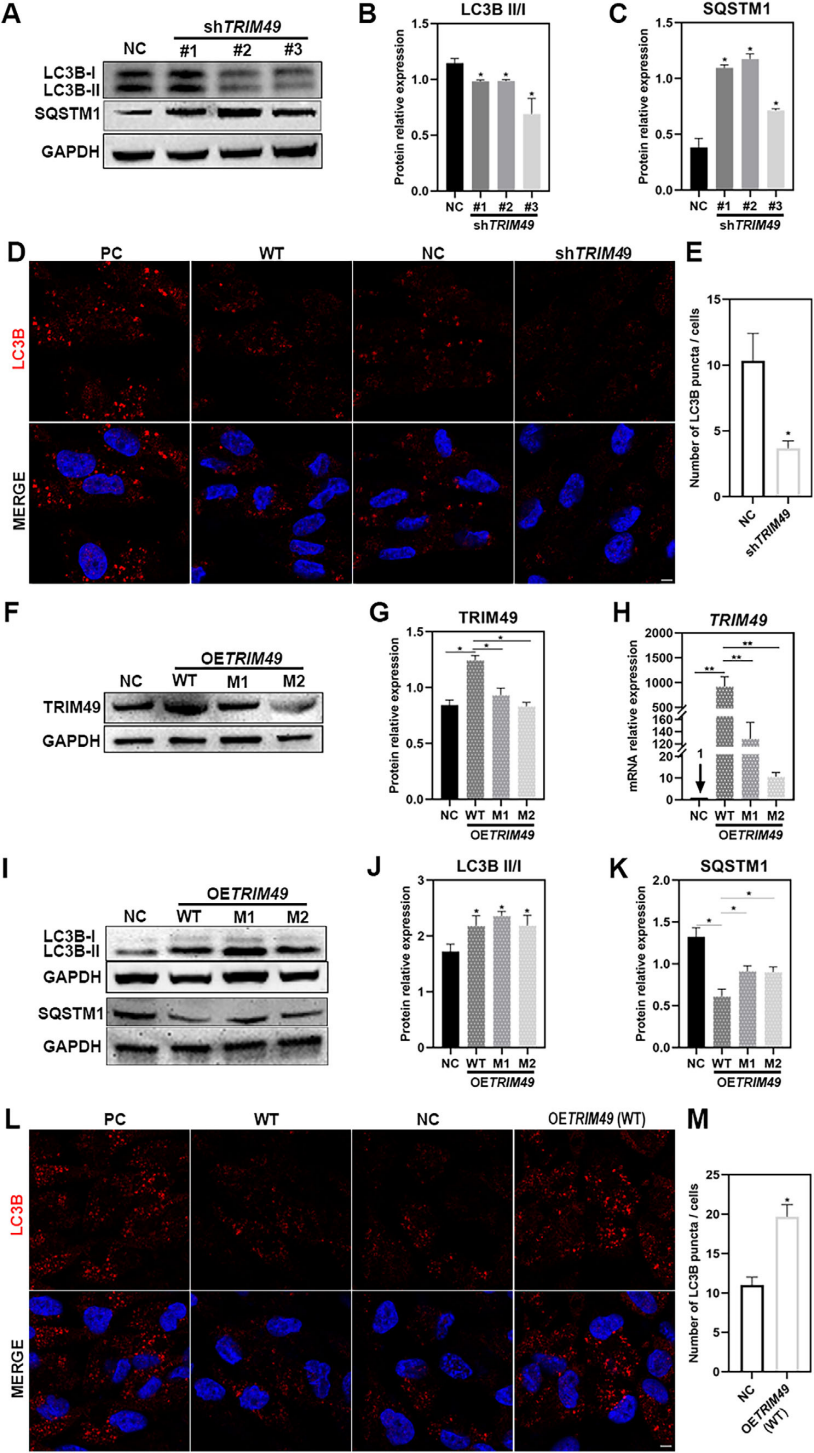
*TRIM49* is involved in maintaining the autophagic flux of human RPE cells. A–C) Immunoblotting and quantification of the expression levels of LC3B and SQSTM1 in normal control (NC) and *TRIM49‐*depleted hTERT RPE‐1 cells. D–E) Immunostaining and quantification of the LC3 puncta in NC and *TRIM49*‐depleted hTERT RPE‐1 cells. The LC3B is stained with red. Starvation‐treated human RPE cells were used as positive controls (PC). Wild‐type (WT) RPE cells without any treatments are used as negative controls. F,G) Immunoblotting and quantification of TRIM49 in NC and wild‐type or mutant (M1 or M2) *TRIM49* overexpression (OE*TRIM49*) hTERT RPE‐1 cells. H) Quantification of *TRIM49* mRNA in NC and wild‐type or mutant (M1 or M2) *TRIM49* overexpression hTERT RPE‐1 cells. *ACTB* was used as a normalizing gene. I–K) Immunoblotting and quantification of the expression levels of LC3B and SQSTM1 in NC and wild‐type or mutant (M1 or M2) *TRIM49* overexpression hTERT RPE‐1 cells. L,M) Immunostaining and quantification of the LC3 puncta in NC and *TRIM49* overexpression hTERT RPE‐1 cells. The LC3B is stained with red. Starvation‐treated human RPE cells were used as positive controls (PC). Wild‐type (WT) RPE cells without any treatments are used as negative controls. *P < 0.05, **P < 0.01. *N* = 3 for each group. Error bars indicate standard deviation.

**Figure 7:**
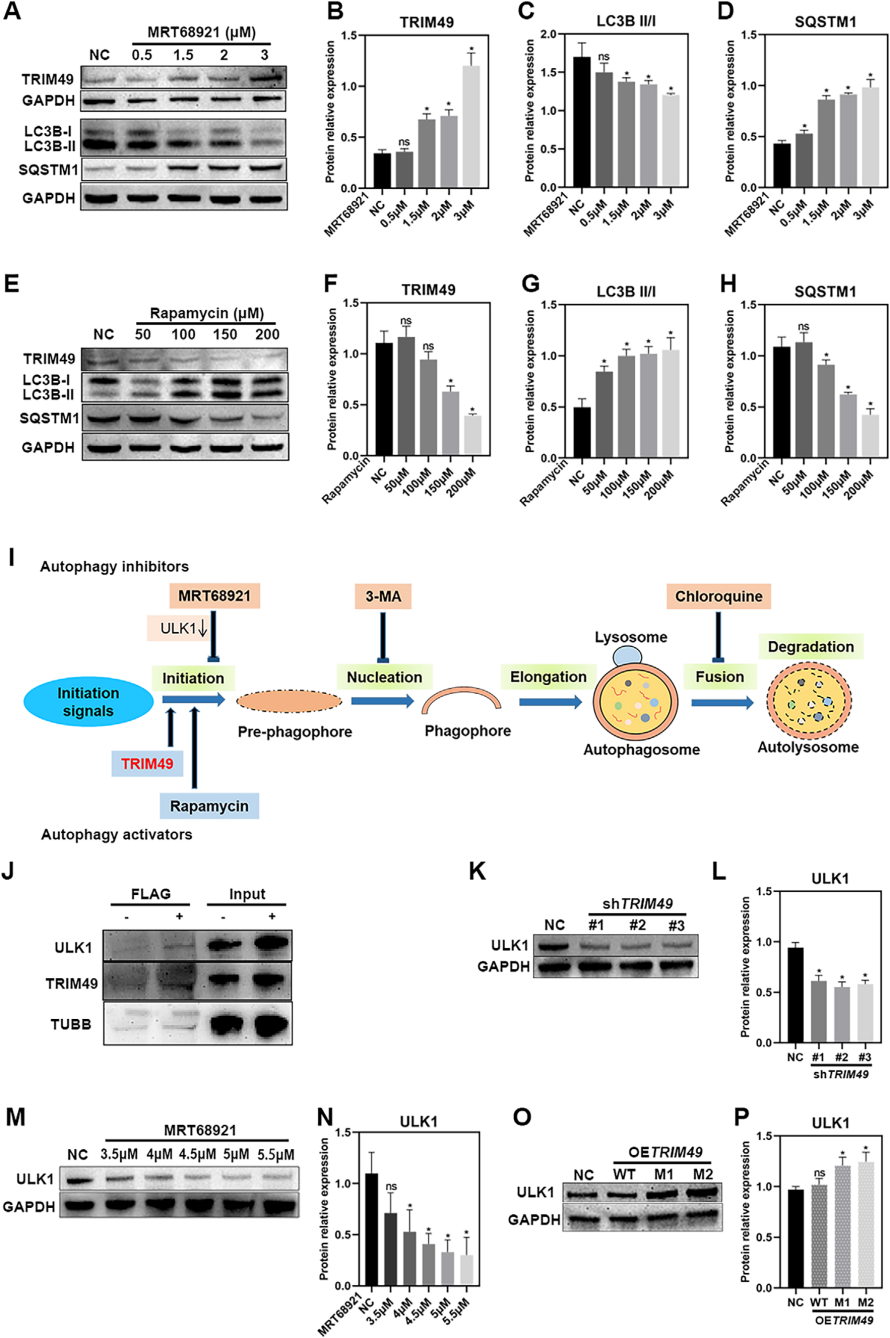
Autophagy modulators lead to changes of *TRIM49* expression levels. A–D) Immunoblotting and quantification of the expression levels of TRIM49, LC3B, and SQSTM1 in normal control (NC) and MRT68921‐treated hTERT RPE‐1 cells. Elevated TRIM49 expression was observed in autophagy–inhibited human RPE cells by MRT68921. E–H) Immunoblotting and quantification of the expression levels of TRIM49, LC3B, and SQSTM1 in NC and rapamycin‐treated hTERT RPE‐1 cells. Reduced TRIM49 expression was observed in autophagy–activated human RPE cells by rapamycin. I) Schematic diagram of autophagic processes and the presumed model of TRIM49 involved in autophagy. J) Co‐immunoprecipitation indicates that TRIM49 interacts with ULK1. K,L) Immunoblotting and quantification of the ULK1 expression in NC and *TRIM49‐*depleted hTERT RPE‐1 cells. M,N) Immunoblotting and quantification of the ULK1 expression in NC and MRT68921‐treated hTERT RPE‐1 cells. O,P) Immunoblotting and quantification of the ULK1 expression in NC and *TRIM49* overexpression (OE*TRIM49*) hTERT RPE‐1 cells. **P < 0.01, ns: no significance. Error bars indicate standard deviation.

**Figure 8:**
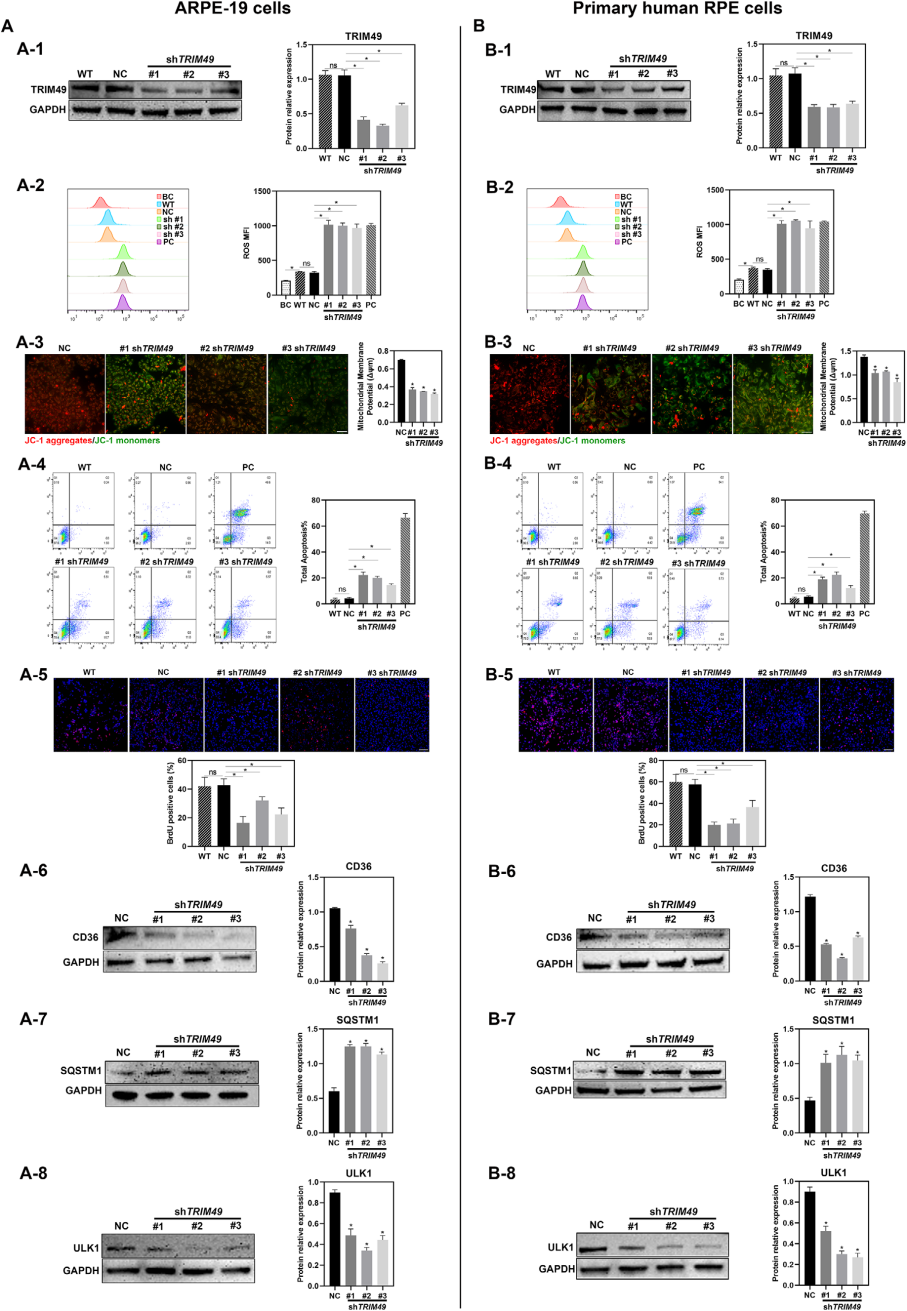
*TRIM49* deficiency disrupts cellular homeostasis and causes decreased phagocytic receptor CD36, autophagic flux, and ULK1 levels in ARPE‐19 cells as well as primary human RPE cells. A) *TRIM49* deficiency in ARPE‐19 cells. (A‐1) Immunoblotting and quantification of TRIM49 proteins in wild‐type, normal control (NC) and *TRIM49*‐depleted ARPE‐19 cells. Elevated levels of ROS (A‐2), decreased Δψm (A‐3), increased apoptosis rate (A‐4), and inhibited cell proliferation (A‐5) are observed in *TRIM49*‐depleted ARPE‐19 cells. JC‐1 aggregates are stained with red, and JC‐1 monomers are stained with green. (A‐6) Immunoblotting and quantification of CD36 expression in NC and TRIM49‐depleted ARPE‐19 cells. (A‐7) Immunoblotting and quantification of SQSTM1 expression in NC and *TRIM49*‐depleted ARPE‐19 cells. (A‐8) Immunoblotting and quantification of ULK1 expression in NC and *TRIM49*‐depleted ARPE‐19 cells. B) *TRIM49* deficiency in primary human RPE cells. (B‐1) Immunoblotting and quantification of TRIM49 proteins in normal control (NC) and *TRIM49*‐depleted primary human RPE cells. Elevated levels of ROS (B‐2), decreased Δψm (B‐3), increased apoptosis rate (B‐4), and inhibited cell proliferation (B‐5) are observed in *TRIM49*‐depleted primary human RPE cells. JC‐1 aggregates are stained with red, and JC‐1 monomers are stained with green. (B‐6) Immunoblotting and quantification of CD36 expression in NC and *TRIM49*‐depleted primary human RPE cells. (B‐7) Immunoblotting and quantification of SQSTM1 expression in NC and *TRIM49*‐depleted primary human RPE cells. (B‐8) Immunoblotting and quantification of ULK1 expression in NC and *TRIM49*‐depleted primary human RPE cells. *P < 0.05, ns: no significance. *N* = 3 for each group. Error bars indicate standard deviation.

**Figure 9:**
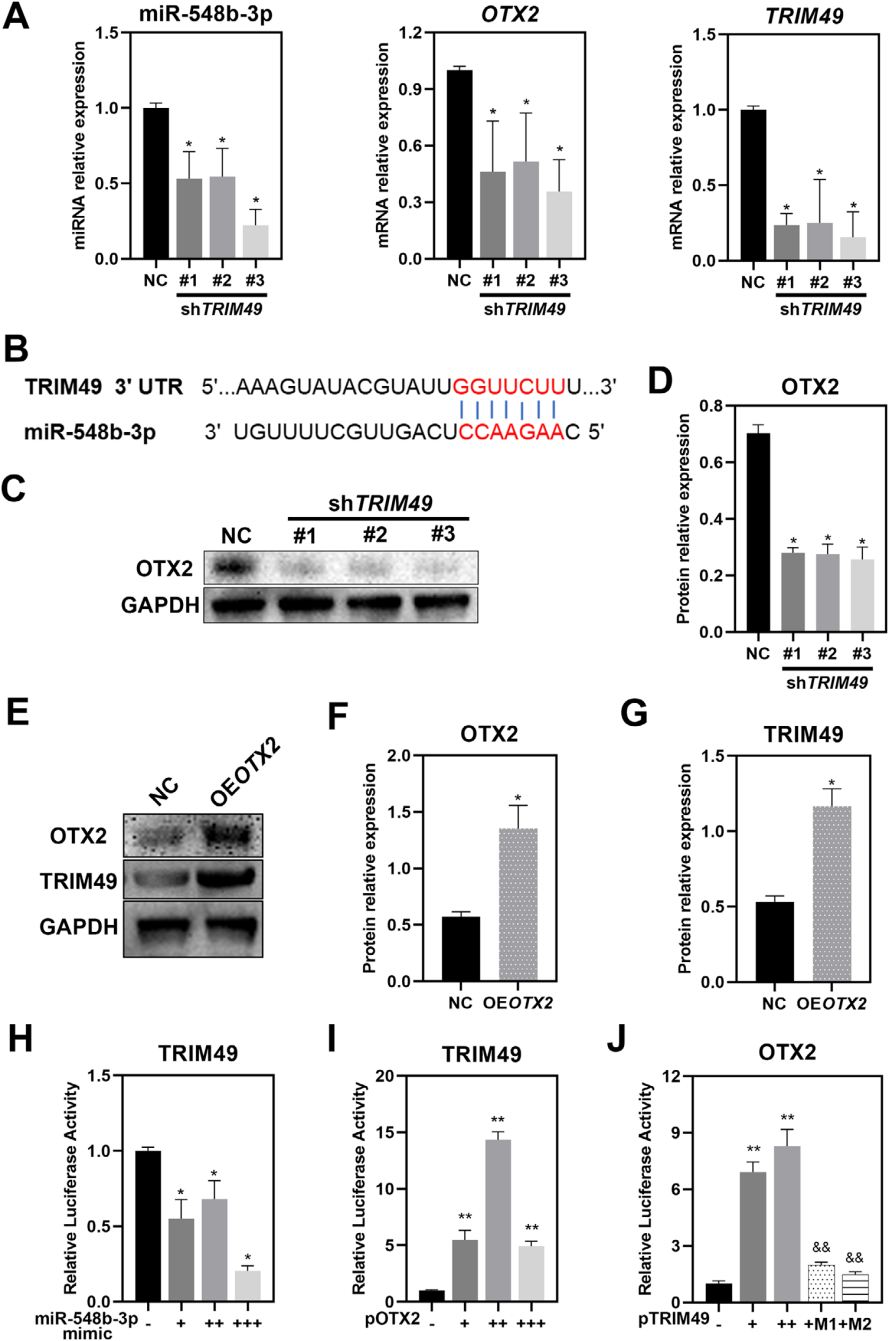
Expression of *TRIM49* is regulated by miR‐548b‐3p/OTX2 pathway. A) Determination of expression of miR‐548b‐3p, *OTX2* mRNA and *TRIM49* mRNA in NC and *TRIM49*‐depleted RPE cells. B) Schematic diagram of the potential binding site between miR‐548b‐3p and TRIM49. C,D) Immunoblotting and quantification of OTX2 expression in NC and *TRIM49*‐depleted RPE cells. E–G) Immunoblotting and quantification of OTX2 and TRIM49 in NC and *OTX2* overexpression hTERT RPE‐1 cells. H,I) Detection of TRIM49 luciferase activity in HEK293T cells co‐transfected with miR‐548b‐3p mimic or p3Flag‐OTX2. J) Detection of OTX2 luciferase activity in HEK293T cells co‐transfected with wild‐type or mutant (M1 or M2) p3Flag‐TRIM49 coding sequence (CDS). *P < 0.05, **P < 0.01, versus the the NC group. ^&&^ *P* < 0.01, versus the wild‐type TRIM49 group. OE: overexpression. *N* = 3 for each group. Error bars indicate standard deviation.

The two unrelated probands with homozygous variants in *TRIM49* had clinical manifestations that met the phenotypic criteria for RP. There was a consanguineous marriage in Family 2 but not in Family 1 (Figure 1A). The parents of the proband from Family 1, F1‐I:1 and F1‐I:2, had normal vision and a normal fundus appearance (**Figure** **2**A; Figure S2A–D, Supporting Information). The proband who is homozygous for the c.1184C>A (p.S395Y) variant (F1‐II:1 in Figure 1A) had experienced nyctalopia since age 10 and reduced visual acuity since age 17. A fundus examination revealed a pale optic disk, attenuated retinal arteries, tapetoretinal degeneration, and atrophy of the RPE (Figure 2B‐2,3; Figure S2E,F, Supporting Information). Fundus autofluorescence (FAF) revealed notably weak fluorescence (Figure 2B‐1), suggesting poor POS phagocytosis by the RPE. Optical coherence tomography (OCT) showed diffuse atrophy of the outer retinal layer, with loss of the ellipsoid zone and much thinner outer nuclear layer (Figure 2B‐5,6) than the normal control (Figure 2B‐7,8). Although the visual acuity is only slightly decreased at 0.6, the visual field is significantly constricted (Figure 2B‐4). Electroretinography revealed undetectable responses in both the rods and cones (Figure 2B‐9). Similarly, the second proband who is homozygous for the c.1134_1137delTCTT (p.L380Gfs*29) variant (F2‐IV:1 in Figure 1A) had experienced night blindness since at least age 30, followed by gradually declining daytime vision. The ophthalmological examination at age 50 revealed attenuated retinal blood vessels, apparent pigmentation in the midperipheral retina, and a wide range of RPE atrophy (Figure 2C‐1,2), consistent with fundus changes seen in progressive RP. A markedly thinned outer retina was observed on OCT (Figure 2C‐3,4). The two RP patients did not complain of any other ocular or related systemic diseases.

### 2.3 Expression and Localization of *TRIM49*

The human *TRIM49* gene, located at 11q14.3, encodes tripartite motif containing 49 (UniProt: P0CI25), an autophagic TRIM interacting with the autophagic factors ULK1, SQSTM1/p62, and LC3.^[^ ^29^, ^35^ ^]^ In humans, *TRIM49* mRNA is abundantly expressed in the retina, followed by the choroid, testis, and brain (**Figure** **3**A). The highest transcription of *TRIM49* in human retina is consistent with the results from GeneCards (GeneCards‐SAGE, https://www.genecards.org/). Among human extraocular tissues, *TRIM49* has the highest transcription level in testis, followed by various brain tissues (Figure 3A). The expression profile is consistent with the Bulk tissue gene expression results from the GTEX consortium (https://gtexportal.org/home/gene/TRIM49), in which there are no expression data on the ocular tissues of human. The localization of the TRIM49 protein in the human retina was evaluated by immunostaining, which shows that it is exclusively located in the RPE of the human retina (Figure 3B). The positional relationships between the localization of TRIM49 and lysosomes were also investigated. In the RPE layer, the staining of TRIM49 partially overlaps with that of the lysosome marker LAMP2 (Figure S3A, Supporting Information). Further subcellular localization analyses reveals that TRIM49 is predominantly located in the cytoplasm and less in the nucleus of RPE cells (Figure 3C; Figure S3B,C, Supporting Information), which is consistent with the localization from GeneCards (https://www.genecards.org/). Together, the expression and localization patterns of TRIM49 are consistent with a potential role in the RPE and are compatible with the RPE dysfunction/atrophy seen in the two RP probands harboring *TRIM49* variants.

### 2.4 *TRIM49* is Essential for Cellular Homeostasis of Human RPE

*TRIM49* is a primate‐specific gene without orthologs in rodents or other common laboratory animals. Stable RPE cell lines provide experimental model systems that possess the same phagocytic machinery as RPE in situ.^[^ ^36^ ^]^ Based on the above considerations and the specific expression of the TRIM49 protein in human RPE cells, we selected hTERT RPE‐1, a stable human RPE cell line, for further cellular and molecular studies. To address the potential role of *TRIM49* in maintaining cellular homeostasis of the RPE, we knocked down (Figure S4A,B, Supporting Information) or overexpressed *TRIM49* (Figure S4C,D, Supporting Information) in hTERT RPE‐1 cells. Compared to wild‐type (WT) and normal control (NC) hTERT RPE‐1 cells (transfected with nontargeting control shRNA), elevated levels of reactive oxygen species (ROS) were present in *TRIM49*‐depleted RPE cells as measured by dihydroethidium (**Figure** **4**A). No significant disparity was observed between the ROS of *TRIM49* overexpressing and NC RPE cells (Figure 4B). *TRIM49*‐depleted RPE cells possess a lower mitochondrial membrane potential (Δψm) as measured by JC‐1 (Figure 4C) and worsened mitochondrial function measured by ATP production (Figure S5, Supporting Information) while *TRIM49* overexpressing RPE cells exhibit a higher Δψm (Figure 4D). The apoptosis rate is highly elevated in *TRIM49*‐depleted RPE cells as measured by annexin V (Figure 4E) but decreased in *TRIM49* overexpressing RPE cells (Figure 4F). In Supplementary materials, gating strategy and unstained control for flow cytometry were displayed (Figure S6A,B, Supporting Information). Inhibited cell proliferation was observed in *TRIM49*‐depleted RPE cells as measured by BrdU incorporation (Figure 4G) while enhanced cell proliferation was present in *TRIM49* overexpressing RPE cells (Figure 4H). In summary, *TRIM49* is essential for maintaining the cell health of human RPE and preventing apoptosis.

### 2.5 *TRIM49* Deficiency Impairs the POS Phagocytosis Capacity of Human RPE Cells and Results in Decreased Phagocytic Receptors

An important function of the RPE is to remove distal POS tips with receptor‐mediated phagocytosis.^[^ ^37^ ^]^ The impairment of POS phagocytosis results in the accumulation of debris in the subretinal space and, thus, photoreceptor degeneration. To ascertain the pathogenicity of *TRIM49* deficiency in retinal degeneration, RPE phagocytic activity was assessed in *TRIM49*‐depleted and NC RPE cells. *TRIM49*‐depleted RPE cells demonstrate significantly fewer ingested fluorescent‐tagged porcine POS compared to NC RPE cells (**Figure** **5**A,B). RPE phagocytic processes comprise three distinct phases—recognition/binding, internalization, and digestion, each of which is separately regulated.^[^ ^16^ ^]^ The fewer POS in RPE cells could result from either dampened internalization or more rapid digestion of POS. Thus, microspheres that can be engulfed by cells but cannot be digested in lysosomes were fed into cultured RPE cells to further delineate the role of TRIM49 in the endocytosis process.^[^ ^38^ ^]^ *TRIM49*‐depleted RPE cells exhibit significantly reduced ingested microspheres compared to NC RPE cells (Figure 5C,D), which indicates dampened target recognition and engulfment in *TRIM49*‐depleted RPE cells. Altogether, these results suggest that *TRIM49* deficiency significantly impairs the phagocytic activity of human RPE cells, which may underlie the formation of subretinal deposits and is crucial for the pathogenesis of retinal degenerative diseases.

Further experiments were performed to investigate the underlying molecular mechanism through which *TRIM49* deficiency affects initiation of POS phagocytosis by the RPE. A series of phagocytic receptors, including MERTK, αv integrin, β5 integrin, and some scavenger receptors (SCARB1, SCARB2, and CD36), were evaluated. Markedly decreased mRNA expression levels are seen for β5 integrin/ITGB5 and the scavenger receptor CD36 in *TRIM49*‐depleted hTERT RPE‐1 cells (Figure S6, Supporting Information). No significant variation or consistent change is observed for MERTK, αv integrin/ITGA5, SCARB1 or SCARB2 in three independent shRNA knockdowns of *TRIM49* (Figure S7, Supporting Information). Both β5 integrin and CD36 were also downregulated at the protein level in *TRIM49*‐depleted RPE cells (Figure 5E,F), consistent with the decreased endocytosis seen in these cells. β5 integrin is one of the primary binding receptors and participates in the first step of POS engulfing by the RPE.^[^ ^19^ ^]^ The scavenger receptor CD36 intervenes in the internalization speed of POS.^[^ ^39^, ^40^, ^41^, ^42^ ^]^ To ascertain the effects of *TRIM49* deficiency on β5 integrin and CD36, the expressions of upstream/downstream molecules of β5 integrin or CD36 were tested in *TRIM49*‐depleted RPE cells. The tetraspanin CD81 is indispensable for the αvβ5 integrin‐dependent particle‐binding step of RPE phagocytosis.^[^ ^43^ ^]^ PTK2/FAK and PGC‐1α/PPARGC1A is involved in β5 integrin‐mediated POS binding by the RPE.^[^ ^44^ ^]^ TLR4, TLR6 and COX2 are downstream molecules of CD36 involved in AMD.^[^ ^45^ ^]^ Compared to NC hTERT RPE‐1 cells, the expression of CD81, PTK2/FAK, TLR4, and TLR6 were noticeably decreased in *TRIM49*‐depleted hTERT RPE‐1 cells (Figure 5G). No significant variation or consistent change is observed for PGC‐1α/PPARGC1A and COX2 in three independent shRNA knockdowns of *TRIM49* (Figure S7, Supporting Information). The phagocytic receptor CD36 has been shown to be associated with autophagic processes in various tissues including the RPE.^[^ ^46^, ^47^, ^48^, ^49^ ^]^ To clarify the relationship between *TRIM49* and CD36 in phagocytosis, sulfosuccinimidyl oleate (SSO), a selective inhibitor of membrane CD36 (Figure S8A, Supporting Information),^[^ ^50^, ^51^, ^52^ ^]^ was used to treat *TRIM49* overexpressing RPE cells. *TRIM49* overexpressing RPE cells demonstrate greater ingested fluorescent‐tagged porcine POS compared to NC RPE cells, and the promotion significantly declined in the presence of SSO in *TRIM49* overexpressing RPE cells (Figure S8B, Supporting Information). Together, these results demonstrated the effects of *TRIM49* on POS phagocytosis by the RPE acting through, or at least involving, CD36‐mediated internalization.

### 2.6 *TRIM49* Regulates the Autophagic Flux of Human RPE Cells

Considering that *TRIM49* as well as the phagocytic receptor CD36 have been associated with autophagic processes, and that autophagy is involved in digesting POS components in the RPE, further experiments were conducted to investigate whether *TRIM49* regulates the autophagic flux of RPE cells. Compared to NC hTERT RPE‐1 cells, the ratio of LC3B‐II to LC3B‐I lessened noticeably, and SQSTM1 was raised in *TRIM49*‐depleted RPE cells (**Figure** **6**A–C). These changes of LC3B and SQSTM1 levels in *TRIM49*‐depleted RPE cells were confirmed by enzyme‐linked immunosorbent assay (ELISA) (Figure S9A,B, Supporting Information). Consistently, the number of LC3B puncta was significantly decreased in *TRIM49*‐depleted cells (Figure 6D,E), indicating that knocking down *TRIM49* suppressed basal autophagy of human RPE cells. To confirm the impact of *TRIM49* on the autophagic flux of human RPE cells, overexpression of wild‐type and mutant (M1 or M2) *TRIM49* was conducted in hTERT RPE‐1 cells (Figure 6F–H). Compared to NC hTERT RPE‐1 cells, the ratio of LC3B‐II to LC3B‐I was markedly heightened, and SQSTM1 was also lessened in wild‐type *TRIM49* overexpression RPE cells (Figure 6I–K). These changes of LC3B and SQSTM1 levels in RPE cells overexpressing *TRIM49* were confirmed by ELISA (Figure S9C,D, Supporting Information). The number of LC3B puncta is significantly increased in *TRIM49* overexpressing cells (Figure 6L,M), indicating that *TRIM49* promotes the autophagic flux of human RPE cells. The promotion significantly declined in RPE cells overexpressing mutant (M1 or M2) *TRIM49* (Figure 6I,K). These results indicate that *TRIM49* regulates the autophagic flux of human RPE cells and that the two variants (M1 and M2) of *TRIM49* disturb this regulation.

### 2.7 *TRIM49* Affects Autophagy via ULK1

To explore the specific autophagic process in which *TRIM49* acts, autophagy in NC hTERT RPE‐1 cells was stimulated by rapamycin treatment and inhibited by chloroquine, 3‐MA, or MRT68921 treatment. Treatment with MRT68921 causes a significant decrease in autophagy and an increase in TRIM49 expression (**Figure** **7**A–D). Similar results are seen with chloroquine treatment (Figure S10A–C, Supporting Information), indicating that the autophagic process in which *TRIM49* participated is upstream of the process in which MRT68921 and chloroquine participate. Conversely, treatment with rapamycin causes a notable increase in autophagy and a drop in TRIM49 expression (Figure 7E–H), indicating that the autophagic process in which *TRIM49* participates is upstream of the point at which rapamycin participates. No changes in autophagy or TRIM49 expression are observed after treatment with 3‐MA (Figure S10D–F, Supporting Information). In combination, the changes in TRIM49 after treatment with the three efficient autophagy modulators indicate that *TRIM49* is involved in the initiation of autophagy (Figure 7I). ULK1 is a key regulator of autophagy initiation^[^ ^53^, ^54^ ^]^ and probably interacts with TRIM49 (Figure 7J). Knockdown of *TRIM49* expression significantly decreases the expression of ULK1 (Figure 7K,L). Reduced ULK1 expression is also present in MRT68921‐treated NC RPE cells (Figure 7M,N).Overexpression of wild‐type *TRIM49* had no impact on the expression of ULK1 (Figure 7O,P). However, a significant increase in ULK1 protein was observed in RPE cells overexpressing the M1 or M2 TRIM49 variant proteins(Figure 7O,P).

To provide additional evidence assessing the validity of hTERT‐RPE1 cell model, we have carried out a number of cellular and molecular experiments in other human RPE cells (**Figure** **8**A,B). We knocked down expression of *TRIM49* in both ARPE‐19 cells (Figure 8A‐1) and primary human RPE cells (Figure 8B‐1). Increased ROS and apoptosis, decreased Δψm and cell proliferation, lessened phagocytic receptors CD36, increased SQSTM1, and reduced ULK1 were observed in both *TRIM49*‐depleted ARPE‐19 (Figure 8A‐2–8) and primary human RPE cells (Figure 8B‐2–8), suggesting that *TRIM49* deficiency disrupts cellular homeostasis and causes decreased phagocytic receptors, autophagic flux, and ULK1 levels in both *TRIM49*‐depleted cell models. Overall, the cellular and molecular impacts of *TRIM49* deficiency on ARPE‐19 and primary human RPE cells are consistent with those on hTERT‐RPE1 cells, confirming the validity of hTERT‐RPE1 cell model.

### 2.8 Expression of *TRIM49* is Regulated by miR‐548b‐3p/OTX2 Pathway in Human RPE Cells

Potential links between *TRIM49* and known IRD genes were also investigated. Potential interacting molecules involved in retinal degeneration or visual cycle, including MINDY3, EPG5, miR‐548b‐3p, OTX2, miR‐519d, LRAT, and NR2E3, were selected based on the STRING and miRDB (MicroRNA Target Prediction Database) databases (Figure S11A,B, Supporting Information). Markedly decreased miR‐548b‐3p and OTX2 mRNA expression levels are seen in *TRIM49*‐depleted hTERT‐RPE1 cells (**Figure** **9**A), while no significant variation or consistent change is observed in MINDY3, EPG5, miR‐519d, LRAT or NR2E3 in three independent shRNA knockdowns of *TRIM49* (Figure S11C,D, Supporting Information). miR‐548b‐3p Was predicted to interact with *TRIM49* and OTX2 based on miRDB (Figure 9B; Figure S11B, Supporting Information). The expression of OTX2 protein also decreased notably in *TRIM49*‐depleted hTERT‐RPE1 cells (Figure 9C,D). To ascertain the interaction between TRIM49 and OTX2, we overexpressed OTX2 in hTERT RPE‐1 cells (Figure 9E,F). Elevated expression of TRIM49 protein is observed in OTX2 overexpressing RPE cells (Figure 9E,G). To corroborate the above findings in human RPE cells, dual‐luciferase reporter assays were performed in HEK293T cells. Following transfection of miR‐548b‐3p mimics, the luciferase activity of TRIM49 significantly decreases (Figure 9H). However, it increases markedly after transfection with the OTX2 coding sequence (CDS) (Figure 9I), in keeping with the results for OTX2 overexpression in human RPE cells. When transfected with wild‐type TRIM49 CDS, the luciferase activity of OTX2 increases notably, while the level of the increase was noticeably attenuated after transfection with variant (M1 or M2) TRIM49 CDS (Figure 9J). These results indicate a coordinated regulatory relationship between TRIM49 and miR‐548b‐3p or OTX2, and the two variants (M1 and M2) in *TRIM49* interfere with this coordination.

## 3 Discussion

Retinal degenerative diseases are one of the leading causes of irreversible vision loss worldwide.^[^ ^55^ ^]^ Dysfunction and damage of the RPE are closely related to the pathogenesis of retinal degenerative diseases, such as age‐related macular degeneration (AMD) and RP.^[^ ^56^ ^]^ Autophagy plays a pivotal role in maintaining RPE function. AMD, one of the most common causes of visual impairment and blindness, is a multifactorial disorder related to aging, genetic and environmental risk factors.^[^ ^57^ ^]^ RP is an inherited retinal degenerative condition, whose causative genes provide promising avenues for dissecting novel autophagy factors in the RPE. Next‐generation sequencing has provided a new way to identify the genetic causes of RP. *TRIM49*, also known as *RNF18*, has previously been associated with autophagic protein degradation.^[^ ^35^ ^]^ To date, the relationship between variants in *TRIM49* and human Mendelian diseases has not been elucidated. In this study, we found two unrelated patients with non‐syndromic RP who were both homozygous for a variant in *TRIM49*. One of the patients was homozygous through an unclear mechanism, possibly a form of UPD. The other was the product of parents who were first cousins. These two variants, c.1134_1137delTCTT (p.L380Gfs*29) and c.1184C>A (p.S395Y), occurring in the SPRY domain of TRIM49, were predicted to be pathogenic by multiple in silico prediction programs. In addition, clinical manifestations that met the clinical criteria for RP and RPE dysfunction and/or atrophy were present in both probands. The highest expression of *TRIM49* mRNA in the human retina and the special expression of *TRIM49* protein in the RPE suggest that it could be important for function of the RPE, a cell type often involved in RP. Moreover, *TRIM49* deficiency leads to dramatically impaired cell health and POS phagocytosis in human RPE cells. These lines of evidence suggest that dysfunction of *TRIM49* contributes to the pathogenesis of some forms of RPE‐associated RP. However, the mechanisms underlying this contribution remain poorly understood.

**Figure 10:**
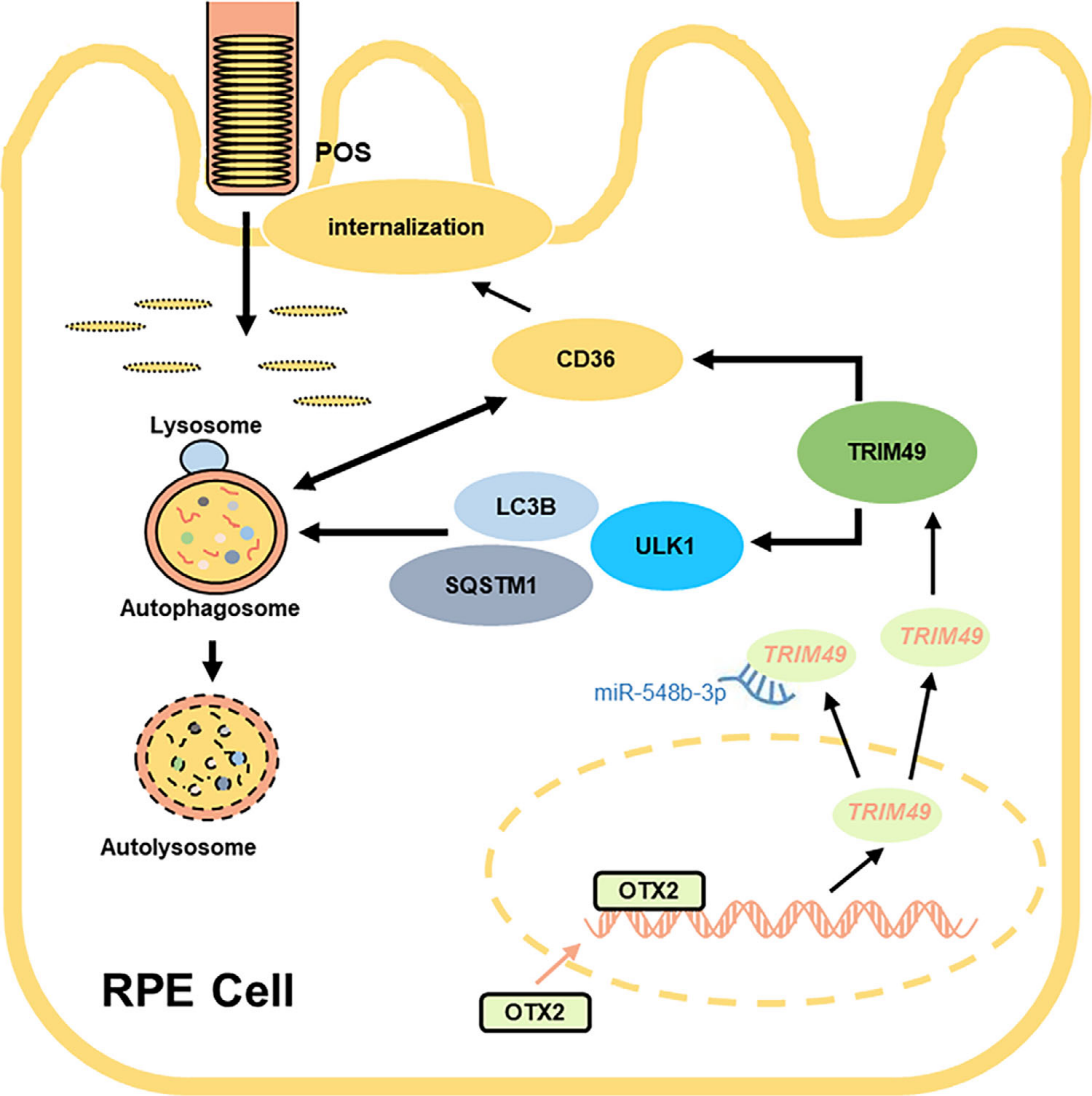
Presumed model of *TRIM49* function in the RPE.

Autophagy supports cell survival and regulates inflammation. Activation of autophagy protects against proapoptotic insults in culture and in vivo in neurodegenerative diseases, where subapoptotic caspase activities may enhance diseases through processes that include the cleavage of mutant proteins to increase their toxicities.^[^ ^58^ ^]^ Prior studies found that the TRIM49 protein plays a role in autophagic protein degradation.^[^ ^35^ ^]^ Specifically, it can regulate autophagy and target autophagic substrates by direct recognition.^[^ ^29^ ^]^ However, the exact role of *TRIM49* in the ocular function was not clear. Here, our data show that *TRIM49* deficiency results in mitochondrial dysfunction of the RPE. Autophagy plays an essential role in selectively degrading damaged mitochondria under mitochondrial toxicity conditions and thus mitochondrial quality control, i.e., mitophagy.^[^ ^59^ ^]^ Deficiency of *TRIM49*, an autophagy factor, may prevent the clearance of damaged mitochondria and result in mitochondrial dysfunction. Moreover, *TRIM49* regulates the autophagic flux of the RPE. From our observations, *TRIM49* is likely to function as an autophagy activator because autophagic flux is strengthened after *TRIM49* overexpression and decreased after *TRIM49* knockdown. Meanwhile, treatments with numerous autophagic regulators indicate that *TRIM49* is involved in the initiation stage of autophagy. Further studies reveal that *TRIM49* interacts with the autophagy initiation protein ULK1 and deficiency of *TRIM49* is associated with decreased ULK1 in human RPE cells. The two *TRIM49* variants seen in our patients disturb the increase in autophagic flux and ULK1 expression in RPE cells overexpressing *TRIM49*, consistent with their pathogenicity. In addition, our data show that *TRIM49* regulates the expression of CD36, a class B scavenger receptor associated with autophagic processes in various tissues.^[^ ^46^, ^47^, ^48^, ^49^ ^]^ The scavenger receptor CD36 intervenes in the internalization speed of POS.^[^ ^39^ ^]^ As one of the phagocytic receptors, CD36 is down‐regulated in *TRIM49*‐depleted RPE cells, which is followed by decreased POS phagocytosis by the RPE, formation of subretinal deposits, and thus photoreceptor degeneration. Taken together, the mechanism through which *TRIM49* deficiency impairs the POS phagocytosis by the RPE involves disruption of CD36‐mediated internalization or autophagy‐mediated protein degradation, or both (**Figure** **10**).

TRIM proteins have emerging roles in the multipronged regulation of autophagy and apoptosis.^[^ ^60^ ^]^ Most TRIMs interact with the BECN1‐ULK1 complex to form TRIMosomes.^[^ ^28^, ^61^ ^]^ They regulate the functions of ATG proteins through physical interactions or ubiquitination.^[^ ^60^ ^]^ In addition, TRIM proteins regulate the activity of MTOR kinase (Mechanistic target of rapamycin kinase) and TFEB (transcription factor EB).^[^ ^62^ ^]^ The subcellular localization and activity of TFEB are regulated by mTOR‐mediated phosphorylation.^[^ ^63^ ^]^ Phosphorylated TFEB is retained in the cytoplasm, whereas dephosphorylated TFEB translocates to the nucleus to induce transcription of target genes. TFEB recognizes E‐boxes present on the promoter of autophagy genes and regulates the expression of ATG (autophagy‐related protein) genes.^[^ ^64^ ^]^ ULK1, also known as ATG1, is one of these ATG genes and is an autophagy‐initiating kinase. *TRIM49* appears to regulate ULK1 expression through modulation of the activity of MTOR kinase and TFEB.

In addition, genes responsible for RP are also involved in multiple molecular pathways and processes, including phototransduction, ciliary transport, and the visual cycle. The visual cycle takes place in rod photoreceptor cells and the RPE.^[^ ^65^ ^]^ Therefore, the molecular mechanisms underlying *TRIM49*‐associated RP were investigated by looking at known IRD genes. In detail, the expression of OTX2 and miR‐548b‐3p are significantly changed in *TRIM49*‐depleted RPE cells, implying an interaction between TRIM49 and OTX2 or miR‐548b‐3p. The two *TRIM49* variants, c.1134_1137delTCTT (p.L380Gfs*29) and c.1184C>A (p.S395Y), markedly decreased this interaction, consistent with their pathogenicity. OTX2 is a transcription factor essential for the development and the maintenance of the RPE.^[^ ^66^ ^]^ miR‐548b‐3p Is one of micro ribonucleic acids (microRNAs) which exert post‐transcriptional regulation via RNA interference and inhibit the expression of their target mRNAs.^[^ ^67^ ^]^ *TRIM49* mRNA was predicted to be the target mRNA of miR‐548b‐3p based on results from the miRDB database (https://www.mirdb.org/, accessed June 2025) and miR‐548b‐3p possesses complementary regions to specific target sites in the 3′ untranslated region (UTR) of *TRIM49* mRNA (Figure 9B). Our data show that the levels of *TRIM49* promoter activity are upregulated by OTX2 but downregulated by miR‐548b‐3p. These results indicate that miR‐548b‐3p and OTX2 may work together to maintain the balance of TRIM49 expression. Considering the effect of *TRIM49* knockdown on the level of *OTX2*, it is proposed that TRIM49 and OTX2 regulate each other. OTX2is a fundamental transcription factor which regulates gene expression in the RPE and neuroretina.^[^ ^68^, ^69^ ^]^ It is probable that *OTX2* regulates the level of *TRIM49* by targeting its promoter region, which contains multiple OTX2 binding sites (JASPAR, https://jaspar.elixir.no/, accessed June 2025). *TRIM49* is essential for mitochondrial function, cell proliferation and cellular homeostasis of human RPE. *OTX2* is a gene that maintains the normal function of RPE cells,^[^ ^66^ ^]^ and its expression level changes in response to cellular states.^[^ ^70^, ^71^, ^72^ ^]^ Thus, *TRIM49* may regulate the level of *OTX2* indirectly through modulation of cell health in the RPE.

Our data also provide new potential targets for therapeutic intervention for retinal degeneration. Previous studies have shown that upregulation of autophagy via mTORC1‐dependent and ‐independent routes enhances the clearance of neurodegenerative disease‐causing proteins and reduces their toxicity.^[^ ^73^, ^74^ ^]^ Activation of autophagy in human RPE cells might allow enhancement of POS phagocytosis, which could be important in the treatment of some RPE‐associated retinal degeneration cases. Augmentation of autophagy‐associated phagocytic receptors such as CD36 may be another potential therapeutic intervention for retinal degeneration. Moreover, investigating the relationship of OTX2 and autophagy in RP pathogenesis would be valuable in further delineating the mechanisms underlying retinal degeneration.

In conclusion, we found that biallelic rare variants in *TRIM49* are associated with autosomal recessive RP. Biochemical investigations have demonstrated a novel role for *TRIM49* in modulating the autophagic flux of RPE cells via ULK1. This autophagic flux regulates cell health and POS phagocytotic capacity of human RPE cells. If *TRIM49* is depleted, the autophagic flux of the RPE is suppressed, leading to impaired POS phagocytosis and the formation of subretinal deposits, followed by drastic photoreceptor degeneration and RP. The link between *TRIM49*‐related autophagy and phagocytosis may be that *TRIM49* deficiency disrupts the autophagic flux of the RPE, which results in decreased CD36 expression, followed by impaired POS phagocytosis. *TRIM49* deficiency thus defines a novel subcategory of retinal degenerations associated with autophagic defects in the human RPE. Although deficiency of some autophagic factors has been shown to cause retinal degeneration in animal studies,^[^ ^24^, ^25^ ^]^ none of their variants previously have been identified in patients with non‐syndromic RP. To the best of our knowledge, this is the first report of possible involvement of autophagic factor *TRIM49* in the blinding retinal degenerative diseases. It is supported by genetic data, cell biological assays, and studies of molecular mechanisms. This study identified not only a novel candidate gene for RP but also a novel autophagic factor in RPE cells. Genetic variants reducing *TRIM49* function lead to suppression of RPE autophagy, which is closely related to the pathogenesis of retinal degenerative conditions such as AMD and RP. Our findings will also provide innovative possibilities for the exploration of new therapies for retinal degenerative diseases.

## 4 Experimental Section

### Human Subjects

Patients with various forms of genetic eye diseases and their available family members were recruited from the Paediatric and Genetic Clinic of the Zhongshan Ophthalmic Center. This study was approved by the Institutional Review Board of the Zhongshan Ophthalmic Center and followed the tenets of the Declaration of Helsinki (2011KYNL012). Written informed consent following the Guidance of Sample Collection of Human Genetic Diseases (863‐Plan) by the Ministry of Public Health of China was obtained from the participants or their guardians before clinical data and peripheral blood samples were collected. Medical and ophthalmic histories, visual acuity measurements, slit‐lamp examination, funduscopic examination, OCT, and ERG were performed. Diagnosis of RP was made by a senior ophthalmologist based on night blindness as the initial symptom, progressive loss of peripheral vision, decreasing visual acuity with age, and fundus changes including waxy pale discs, retinal arteriolar attenuation, tapetoretinal degeneration or pigment deposits in the midperipheral retina. The pedigrees were drawn using questionnaire or oral description.

### WES and Bioinformatic Analysis

WES was performed on genomic DNA from the two probands with RP, as well as 7283 unrelated individuals with other ocular conditions, which include 492 unaffected individuals (i.e., normal controls), 1561 probands with high myopia, 1231 probands with glaucoma, and 3999 probands with other genetic eye diseases. The procedures for sequencing, variant calling and annotation, and the filtering of potentially pathogenic variants by multistep bioinformatic analyses were performed as previously described.^[^ ^9^ ^]^ Samples with pathogenic variants in known RP genes were excluded from this study. Unique biallelic rare variants were searched in WES data from the two probands with RP and then compared with in‐house data from 7283 controls and the gnomAD database. The raw WES data of the two probands had been deposited in the Genome Sequence Archive (Genomics, Proteomics & Bioinformatics 2021) in the National Genomics Data Center (Nucleic Acids Res 2022), China National Center for Bioinformation / Beijing Institute of Genomics, Chinese Academy of Sciences (GSA‐Human: BAT00430) that are publicly accessible at https://ngdc.cncb.ac.cn/gsa‐human.^[^ ^75^, ^76^ ^]^

### Sanger Sequencing

Sanger sequencing was conducted to validate the candidate variants in the probands. Segregation in available family members was further evaluated. Primers (Table S3, Supporting Information) were designed using Primer3 (http://primer3.ut.ee/) and used to amplify genomic fragments harboring variants. The procedures used for amplification, sequencing, and target fragment analysis were performed as previously described.^[^ ^9^ ^]^

### Human Ocular Tissues

Human ocular tissues were obtained from the Eye Bank of Guangdong Province, and were from an eye donor who died of meningioma. All procedures conformed to the Declaration of Helsinki developed by the World Medical Association and the ethics principles of the International Ethical Guidelines for biomedical research involving human subjects developed by the Council for International Organizations of Medical Sciences. Written informed consent was obtained from the donor's family prior to the study. This study was approved by the lnstitutional Review Board of the Zhongshan Ophthalmic Center, Sun Yat‐sen University, Guangzhou, China (2023KYPJ200).

### Quantitative Real‐Time PCR (qPCR)

RNA samples were prepared from 31 human tissues or cultured cells. The 31 human tissues included 24 extraocular samples purchased from Clontech Laboratories (CA, USA) and seven ocular tissues from the eye donor described above. The RNA samples from 24 human extraocular tissues were pooled from 12 male and female Caucasian individuals who experienced sudden death at the age of 18 to 54 years. cDNA Was synthesized from the total RNA using the PrimeScript RT Reagent Kit (RR047A, TaKaRa, Japan). All primer sets used for qPCR (Table S4, Supporting Information) were designed using Primer 3 (http://primer3.ut.ee/). qPCR Was performed with PowerUp SYBR Green Master Mix (A25742, Applied Biosystems), and the fold changes in RNA levels were calculated using the ΔΔCt method, as described in a previous study.^[^ ^77^ ^]^ The primers were synthesized by Tsingke Biotech (Beijing, China).

### Immunohistochemical Staining

Paraformaldehyde‐fixed human eyes were embedded in paraffin and cut into 4‐µm‐thick sections. The sections were subjected to antigen retrieval at 98 °C for 30 min and subsequently blocked with 1% bovine serum albumin. Staining for TRIM49 in human retina was performed with a rabbit anti‐TRIM49 antibody (Invitrogen Cat# PA5‐31431, epitope between residues 1 and 272), a mouse anti‐RPE65 antibody (Invitrogen Cat# MA1‐16578), a mouse anti‐rhodopsin 1D4 antibody (Abcam Cat# ab5417), a mouse anti‐LAMP2 antibody (Abcam Cat# ab25631), and a rabbit anti‐opsin red/green antibody (Millipore Cat# AB5405) at final dilutions of 1:100, 1:500, 1:1000, 1:100, and 1:500, respectively. Secondary antibodies included AlexaFluor 555‐conjugated goat anti‐rabbit IgG antibodies and AlexaFluor 488‐conjugated goat anti‐mouse IgG antibodies (Cell Signaling Technology Cat# 4413; Cell Signaling Technology Cat# 4408, both antibodies were used at a final dilution of 1:500). Then, sections were treated with TrueBlack Lipofuscin Autofluorescence Quencher for 30 s. Images of stained sections were captured using a confocal microscope (LSM980, Carl Zeiss, Germany; C2, Nikon, Japan).

### Cell Culture

The hTERT‐RPE1 cell line was purchased from ATCC (CRL‐4000, Lot# 70021355). Cells were cultured, subcultured and maintained strictly following ATCC instructions. The cells were cultured in DMEM:F12 Medium (Gibco, Thermofisher Scientific, USA) with 10% FBS (Gibco, Thermo Fisher Scientific, USA) and 0.01 mg ml^−1^ hygromycin B (400052, Merck Millipore) at 37 °C with 5% CO_2_. The characteristics of hTERT‐RPE1 cells were validated by detecting the expression of the RPE‐specific markers RPE65, pancytokeratin, ZO‐1, and BEST1 in both original and clonal ones (Figure S12A–D, Supporting Information). For all four markers, the staining patterns in the clonal ones were similar to those in the original ones. The polarized status of RPE cells were checked by detecting the localization of the RPE basolateral markers BEST1 and collagen IV (Figure S13A,B, Supporting Information). For both markers, the localization in RPE includes from the basal to the apical, indicating that the RPE cells were not polarized.

ARPE‐19 (Adult retinal pigment epithelial cell line‐19, ATCC, Virginia, USA) cells were obtained from the American type culture collection. The cells were cultured in high glucose Dulbecco's modified Eagle's medium (DMEM, HyClone, GE Healthcare Life Sciences, United States) with 10% FBS (Gibco, Thermo Fisher Scientific, United States) and 1% of penicillin/streptomycin (HyClone, GE Healthcare Life Science, United States) at 37 °C with 5% CO_2_.

Primary human RPE cells were isolated from the eyeballs of a donor who died accidentally without ophthalmic diseases, who was 43 years old, from the Eye Bank of Guangdong Province (Zhongshan Ophthalmic Center, Sun Yat‐sen University), adhering to the tenets of the Declaration of Helsinki. The isolation and culture of primary‐RPE cells were performed as described previously.^[^ ^78^ ^]^ The isolated primary human RPE cells were cultured in a Petridish lined with BD Matrigel substrate membrane matrix. The culture medium contained 20% fetal bovine serum (GIBCO, Life Technology, United States) and 1% penicillin (100 µG mL^−1^) and 1% streptomycin (100 µ DMEM/f12 (hyclone, GE Healthcare Lifescience, United States).

The RPE cells were used when 80–90% confluent (Figure S12C,D, Supporting Information) except for quantifying autolysosomes, which requires a lower density to provide a clearer view of staining puncta. All assays were conducted before 30 passages.

### Plasmids and Transfection

For *TRIM49* knockdown, hTERT‐RPE1 cells, as well as ARPE‐19 and primary human RPE cells, were transfected with pLKO.1‐TRC negative control vectors and sh‐TRIM49 plasmids (Table S5, Supporting Information) using Attractene Transfection Reagent (QIAGEN, German, Dusseldorf) following the manufacturer's instructions. Lentiviral transfection was performed as described previously.^[^ ^79^ ^]^ Briefly, cells (2×10^5^/mL) were seeded in 6‐well plate and then were infected with the lentiviruses. Polybrene (10 µg mL^−1^) (Sigma, USA, New York) was added to the lentiviruses to enhance infection efficiency. Pooled stable populations of hTERT‐RPE1 cells were generated by treatment with puromycin (10 mg mL^−1^; Thermo Fisher Scientific, USA, New York) for 48 h. All plasmids used in the paper were synthesized by Kidan Biosciences co.Ltd (Guangzhou, China). To transfer plasmids containing wild‐type or mutant TRIM49, wild‐type OTX2, and wild‐type NR2E3 CDS into hTERT‐RPE1 cells, the cells were seeded on either 24 or 96‐well cell culture‐treated plates at a density to ensure 80% confluent cultures at 24 h after seeding. Typically 10^5^ cells cm^−2^ surface area of the culture dish were seeded in cell‐specific medium. Transfection using Lipofectamine 3000 was performed according to the manufacturer's protocol with a DNA to Lipofectamine ratio of 1:3 w/v. A transfection enhancer, the 3000 enhancer reagent (1:2, DNA:Reagent, w/v), was used along with the Lipofectamine 3000 transfection reagent for all transfections. Typically, 100 or 500 ng of plasmid DNA were transferred to each well of the 96‐well and 24‐well plates, respectively. Chloroquine (S6999), MRT68921 HCl (S7949), 3‐MA (S2767), and Rapamycin (S1039) used to treat hTERT‐RPE1 cells were all purchased from Selleck.

### Cellular Oxidative Stress Assay

ROS were measured with Dihydroethidium (DHE) (Beyotime, China) by flow cytometry. After collected from 6‐well plates, cell samples were prepared in 1× Assay Buffer at 1 × 10^6^ cells mL^−1^. After this, 10 µL of the cell sample were incubated with 190 µL of DHE working solution under 37 °C in the dark for 30 min before being run on the BD LSRFortessa instrument (BD Biosciences, Franklin Lakes, NJ, USA).

### Mitochondrial Membrane Potential (ΔΨm) and ATP Assays

ΔΨm staining was performed using the Enhanced mitochondrial membrane potential assay kit with JC‐1 (Beyotime, China), 5000 cells/well were seeded in 96‐well plates (Corning, United States), and 100 µL of JC‐1 solution was added to the cells for 20 min. When the ΔΨm is high, JC‐1 collects in the mitochondrial matrix to form polymers (JC‐1 aggregates), which could produce red fluorescence. When the ΔΨm was low, JC‐1 could not gather in the mitochondrial matrix, and JC‐1 was a monomer (JC‐1 monomers) and could produce green fluorescence. The maximum excitation and emission wavelength of JC‐1 monomers were 514 and 529 nm, respectively. The maximum excitation and emission wavelength of JC‐1 aggregates are 585 and 590 nm, respectively. The absorbance at 490/530 and 525/590 nm was detected using a microplate reader (BioTek Instruments, Winooski, Vermont, United States). Images of stained cells were captured using a fluorescence microscopy (Eclipse Ni, Nikon, Japan).

The ATP content was determined by using an Enhanced ATP Assay Kit, S0027 (Beyotime Biotechnology, Shanghai, China) according to the manufacturer's instructions. The concentration of ATP was calculated according to an ATP standard curve and expressed as µmol OD_730_^−1^.

### Cellular Apoptosis Analysis and Cell Proliferation Measurements

Apoptosis was examined by flow cytometric analysis. An annexin V stain assay (Merck Millipore, Germany) was performed following the manufacturer's protocol. Cells were seeded in 6‐well plates at a density of 1 × 10^5^ cells per well. After treatments, cells were separated from the wells and centrifuged. After being re‐suspended, the cells were subjected to MUSE Annexin V & Dead Cell Kit and incubated at room temperature in the dark for 20 min. Then, the cell suspensions were mixed thoroughly and analyzed using a BD LSRFortessa instrument (BD Biosciences, Franklin Lakes, NJ, USA). The acquired data were processed using FlowJo 10.0 (FlowJo Co., Ashland, OR, USA).

Cell proliferation was measured by BrdU staining. BrdU (5‐Bromo‐2‐deoxyUridine, Servicebio, G4102) was added to the media of growing cells at 100 µm for 4 h. The cells were then washed with phosphate‐buffered saline (PBS), fixed with 4% paraformaldehyde solution (20 min). Cells were imaged following application of standard immunofluorescence protocols: Triton 100‐X solution permeabilization, 1 M HCl addition, anti‐BrdU primary antibody addition (1:100, Servicebio, G4102), anti‐mouse AlexaFluor 594 (1:500, Abcam), and DAPI nuclear staining. Images of stained cells were captured using a fluorescence microscopy (Eclipse Ni, Nikon, Japan).

### Phagocytosis Assays

Cells were challenged by either FITC‐labeled porcine POS or microspheres (Fluoresbrite Yellow Green Microspheres, 1.00 µm, green fluorescent (441/486), Polysciences, Cat#: 17154). The procedure of porcine POS isolation and labeling was as described previously.^[^ ^80^ ^]^ For porcine POS treatment, cells were incubated with porcine POS for 2 and 6 h at a density of 10 POS/cell. Cells were then subjected to a standard immunofluorescence protocol and mounted for imaging. For microsphere treatment, cells were incubated with microspheres for 2 and 6 h at a density of 10 microspheres/cell, and then imaged as described above. The numbers of engulfed POS or microspheres per RPE cell were counted and quantified using Image J software.

### Dual‐Luciferase Reporter Assay

Three luciferase reporter vectors, TRIM49, OTX2, and NR2E3, were constructed to analyze dual luciferase reporter genes in HEK293T cells. Promoter plasmids were transfected into HEK293T cells. After 24 h, cells were treated with p3Flag‐TRIM49‐wt, TRIM49‐mut1/2, NR2E3, OTX2 or vehicle, for another 24 h. A dualluciferase reporter assay kit (Promega; E1910) was used to determine luciferase activity. Relative luciferase activities were calculated by normalizing firefly luciferase activity values to respective Renilla luciferase values.

### Autophagic Flux Determination in RPE Cells

The hTERT‐RPE1 cells were plated in 6‐well plates. Autophagic flux was blocked by treating cells with 80 nm bafilomycin A1 (Selleck, S1413) in the presence of complete growth medium for the same period for 3 h. The coverslips were incubated with a rabbit anti‐LC3B antibody (L7543, Sigma) at a final dilution of 1:500. LC3 puncta were examined with Zeiss LSM880 confocal microscope (Carl Zeiss, Germany). The number of LC3 puncta was counted using Image J software.

### Western Blotting and Enzyme‐Linked Immunosorbent Assay (ELISA)

Total protein concentration was determined using a bicinchoninic acid (BCA) assay (cat# 23227, BCA Protein Assay Reagent Kit, Thermo Fisher Scientific Pierce Protein Research Products, Rockford, IL). The western blotting assay was conducted as previously described.^[^ ^81^ ^]^ A full list of primary antibodies and dilutions used for western blotting is shown in Table S6 (Supporting Information). Results were representative of at least 3 experiments. The concentrations of LC3B and SQSTM1 in the RPE cells were measured by sandwich ELISAs using human LC3B (JINGMEI, SEJM‐6516H2) and SQSTM1 JINGMEI, SEJM‐1393H2) ELISA kits according to the manufacturer's instructions. Briefly, the RPE cells were centrifugated to remove particulates. This was followed by performing the ELISA in 48‐well microtiter plates coated with cell culture supernates and incubated for 60 min at 37 °C. The reaction was stopped with 50 µl Stop Solution in each well, and absorbance was measured at 450 nm using a microplate reader.

### Immunofluorescence

For cell slides, 1 × 10^5^ of cells were first grown on coverslip in each well of 24‐well plates. After treatments, the cell slides were fixed with 4% paraformaldehyde solution (Beyotime, China) for 15 min. Then, the coverslips were penetrated with 0.5% of Triton 100‐X solution (Sigma‐Aldrich, USA) for 10 min and blocked with Normal Goat Serum (Solarbio Science & Technology, China) for 1 h at room temperature, the coverslips were incubated with a rabbit anti‐RPE65 antibody (ab231782, Abcam) at a final dilution of 1:50, a mouse anti‐pancytokeratin antibody (53‐9003‐82, Invitrogen) at a final dilution of 1:50, a rabbit anti‐ZO‐1 antibody (61‐7300, Invitrogen) at a final dilution of 1:500, and a rabbit anti‐BEST1 antibody (ab259836, Abcam) at a final dilution of 1:500. On the second day, the coverslips were incubated with AlexaFluor 488‐conjugated goat anti‐mouse IgG antibodies (ab150113, Abcam), or AlexaFluor 594‐conjugated goat anti‐rabbit IgG antibodies (ab150080, Abcam) at a final dilution of 1:500 for 1 h at room temperature in darkroom. Nuclei were counterstained with DAPI (Beyotime, China) for 10 min at room temperature and mounted with Antifade Mounting Medium (Beyotime, China). Images were later captured using a confocal microscope (LSM880 or LSM980, Carl Zeiss, Germany). Images were processed and viewed with ZEN blue (v2.6, Carl Zeiss, Germany) software.

### Statistical Analysis

Data were analyzed in GraphPad Prism 10.1.1. Mann‐Whitney U‐test was made to calculate significant differences between different experimental conditions. The protein density was analyzed using Image J software, and both protein and mRNA expression levels were compared between *TRIM49* knockdown, overexpression, and a NC group. Statistical significance was defined as a *p*‐value less than 0.05 (*p* < 0.05).

## Conflict of Interest

The authors declare no conflict of interest.

## Author Contributions

Z.Y., C.W., H.D., and Y.W. contributed equally to this work. Z.Y., C.W., H.D., Y.W., W.S., H.S., and Q.Z. designed the study. W.S., J.W., X.X., S.L., X.J., P.W., and Q.Z. recruited patients. Z.Y., Y.W., S.Z., J.W., X.X., Y.J., and J.O. collected the clinical data. X.X., S.L., and Q.Z. performed whole exome sequencing. Q.Z., Z.Y., S.L., and P.W. performed the bioinformatic analysis. Z.Y. and J.W. constructed 3D models of wild‐type and mutant TRIM49 proteins. Z.Y. and Y.W. performed the expression analysis of *TRIM49* mRNA. Z.Y. performed the localization analysis of *TRIM49* protein. Z.Y., C.W., H.D., Y.W., S.C., J.L., and S.Z. conducted the cellular and molecular experiments. Z.Y., C.W., H.D., and Y.W. analyzed the experimental data. Z.Y., C.W., H.D., Y.W., W.S., H.S., and Q.Z. discussed the results. Z.Y., C.W., H.D., and Y.W. wrote the manuscript. J.F.H., W.S., and Q.Z. revised the manuscript. All authors reviewed and approved the manuscript.

## Supporting information



Supporting Information

## Data Availability

The data from WES reported here were deposited in the China National Center for Bioinformation (BAT00430) database that is publicly accessible at https://ngdc.cncb.ac.cn/gsa‐human.
