# Supplementary material for: Retinitis Pigmentosa‐Associated Gene TRIM49 Regulates ULK1‐Mediated Autophagy and Photoreceptor Phagocytosis by the Retinal Pigment Epithelium
Source: Adv Sci (Weinh). 2025 Sep 16;12(43):e12305. doi: 10.1002/advs.202512305 (PMC12631925; doi:10.1002/advs.202512305)
Supplement: Supplementary file 1 — Supporting Information [file ADVS-12-e12305-s001.docx]

**Supplementary figures**


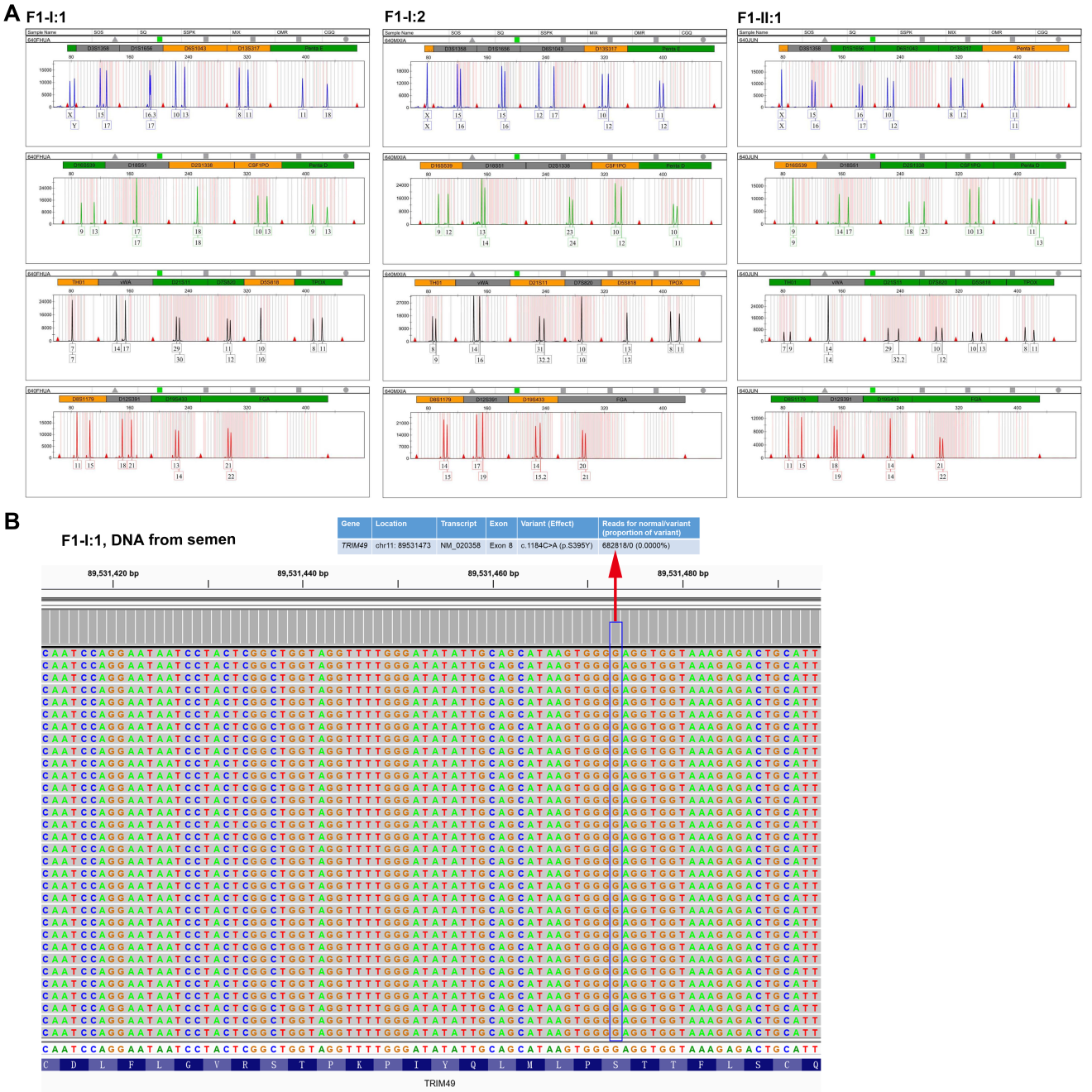


Fig. S1. Sequence chromatogram of 20 STR markers and Ultra-high-depth next-generation sequencing in Family 1. (A) Sequence chromatogram of 20 STR markers in F1-I:1, F1-I:2, and F1-II:1, confirmed that the presumed father F1-I:1 and presumed mother F1-I:2 are the biological father and mother of F1-II:1, respectively. (B) Ultra-high-depth next-generation sequencing (20,000×, 682,818 reads) of DNA from F1-I:1’s semen revealed the nucleotide in g.89531473 is G for 100% and none T was detected, suggesting no mosaicism of the variant c.1184C>A (p.S395Y) in F1-I:1.


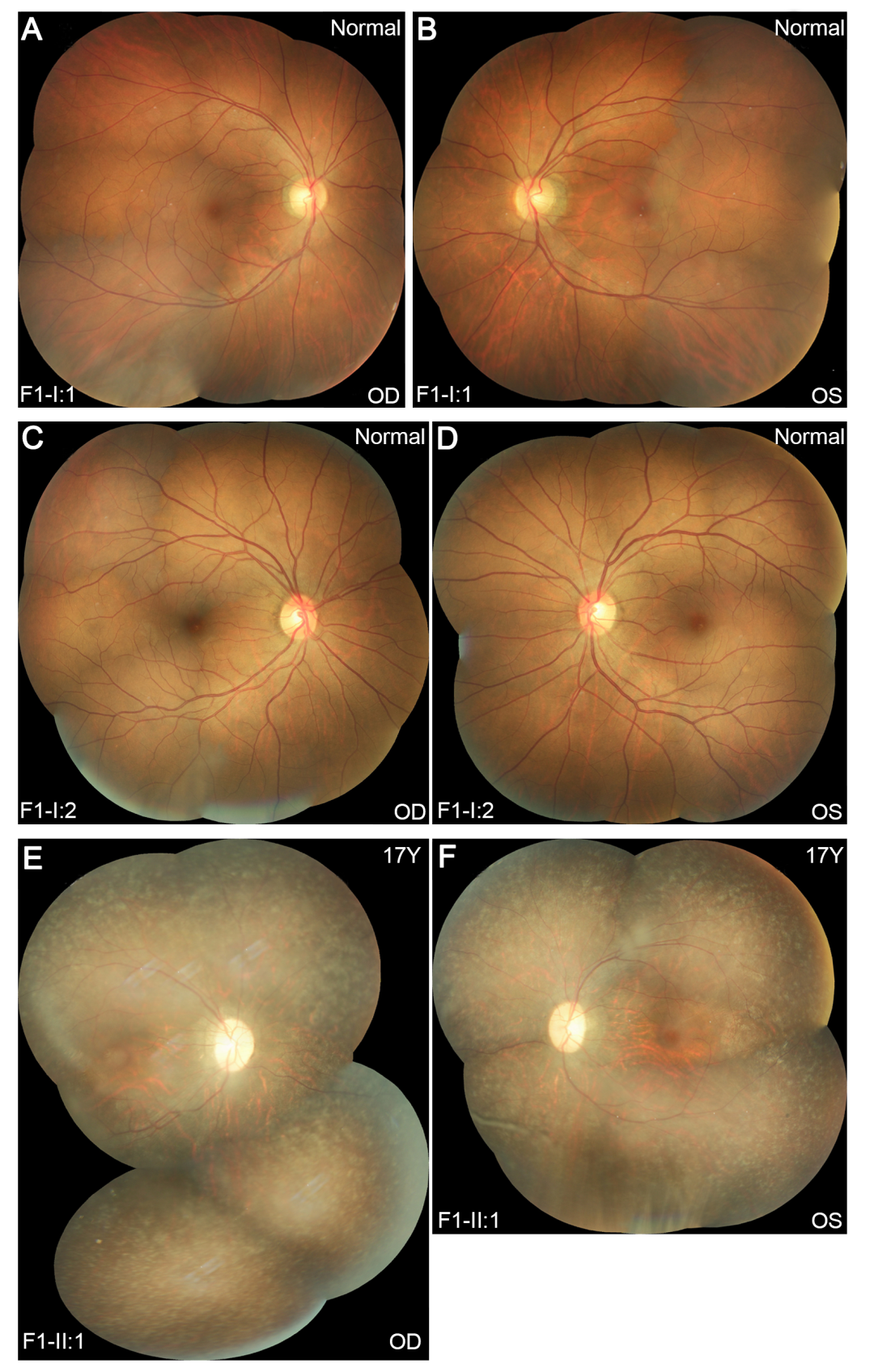


Fig. S2. Wide-field fundus photography of individuals from Family 1. (A–D) Normal fundus photographies are present in unaffected individuals F1-I:1 and F1-I:2. (E–F) Wide-field fundus photography from the proband F1-II:1 at age 17 years. A pale optic disc, attenuated retinal blood vessels, tapetoretinal degeneration, pigmentary disorders, and atrophy RPE in posterior pole are observed. OD, right eye; OS, left eye; Y, years.


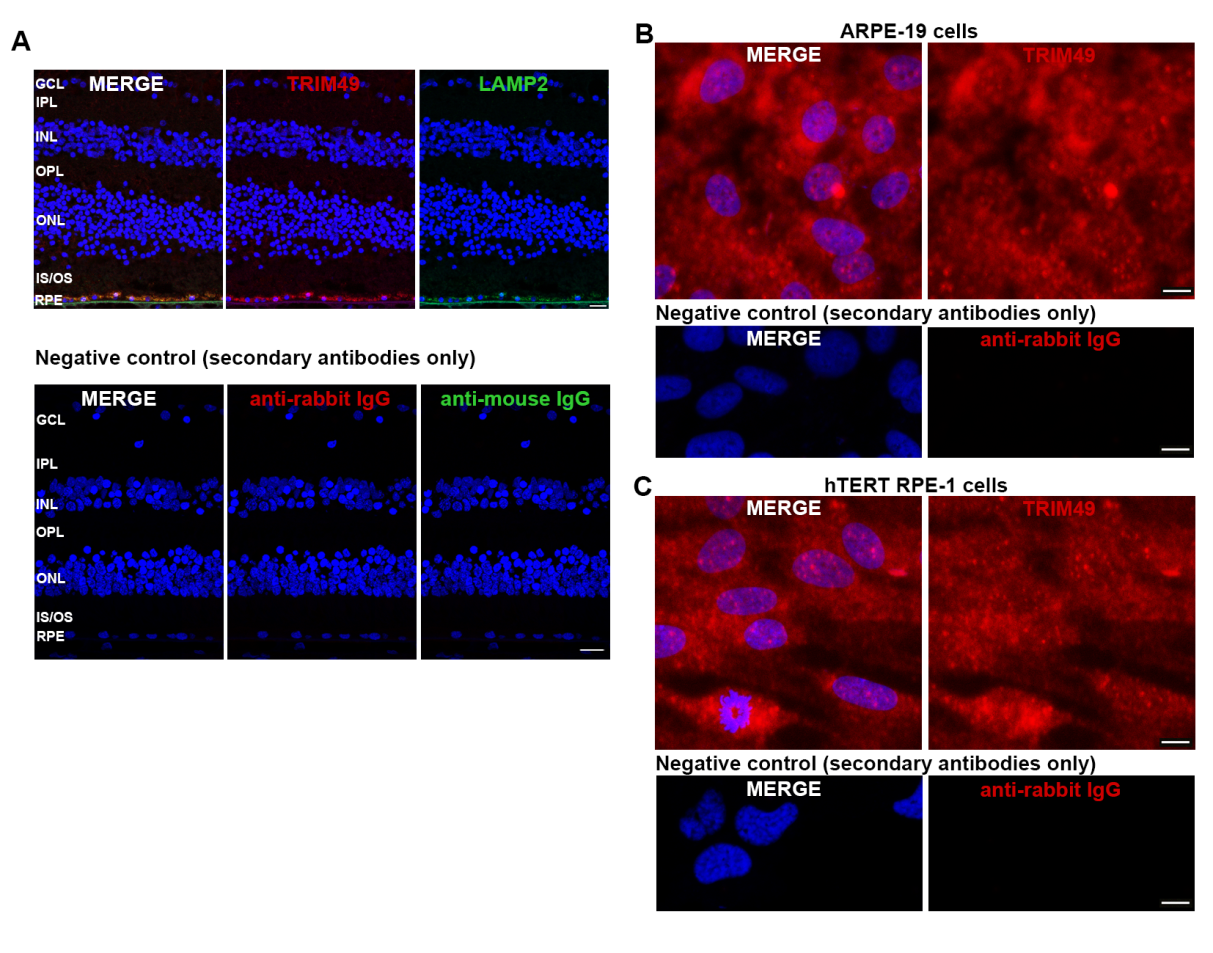


Fig. S3. Immunostaining of TRIM49 in the human retina and RPE cells. (A) Staining of TRIM49 (red) and LAMP2 (green) in human retina. GCL, ganglion cell layer; IPL, inner plexiform layer; INL, inner nuclear layer; OPL, outer plexiform layer; ONL, outer nuclear layer; IS, inner segments of photoreceptors; OS, outer segments of photoreceptors; RPE, retinal pigment epithelium layer. The scale bar represents 20 μm. (B) Staining of TRIM49 (red) in ARPE-19 cells. The scale bar represents 10 μm. (C) Staining of TRIM49 (red) in hTERT RPE-1 cells. The scale bar represents 10 μm.


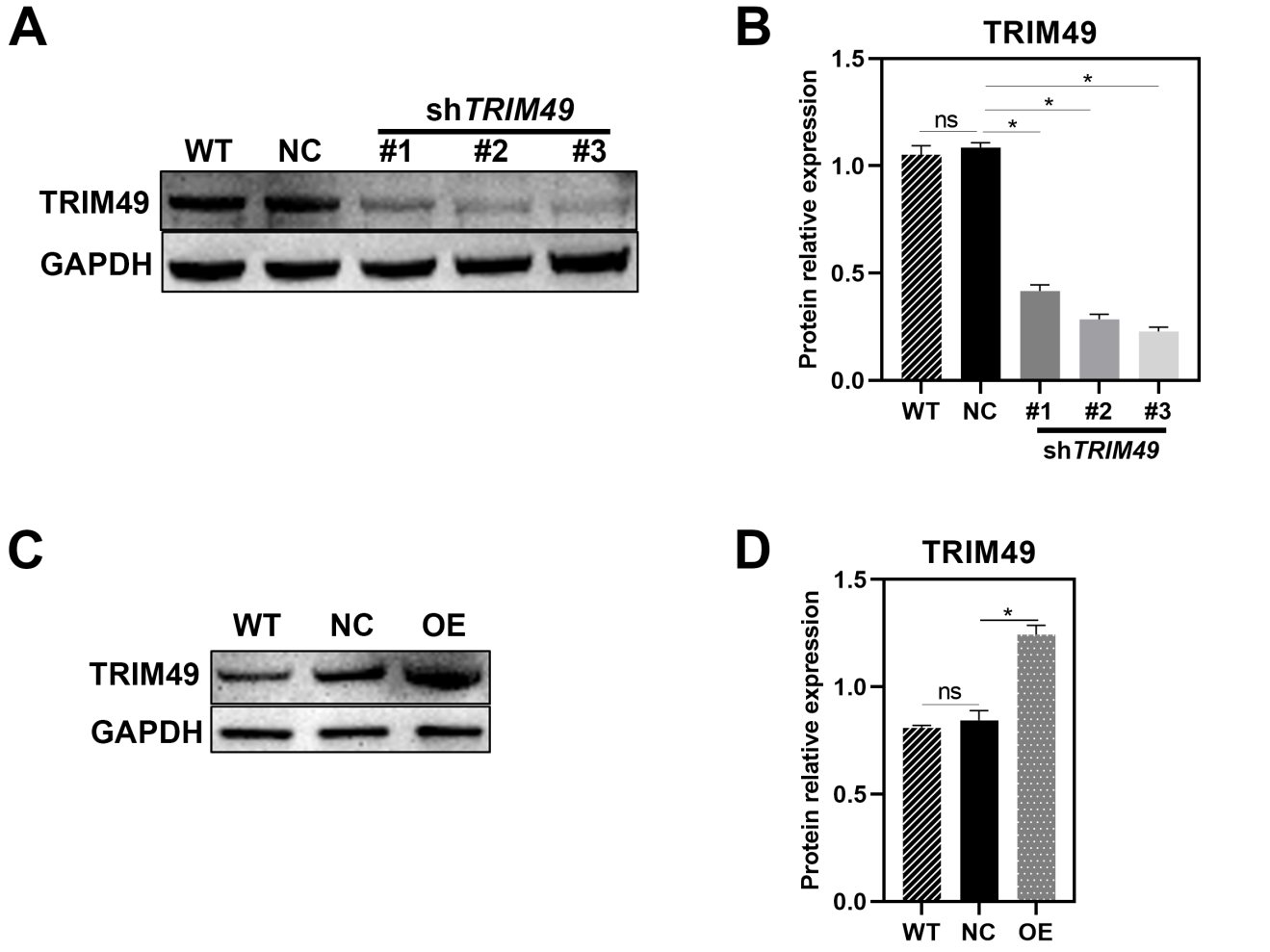


Fig. S4. Detection of the interfering effect of the shRNA and CDS against TRIM49 in hTERT RPE-1 cells. (A) Immunoblotting of TRIM49 in wild-type (WT), normal control (NC) and TRIM49-depleted hTERT RPE-1 cells. (B) Quantification of TRIM49 proteins presented in panel A. Relative expression of TRIM49 in relation to GAPDH. (C) Immunoblotting of TRIM49 in WT, NC and TRIM49 overexpression (OE) hTERT RPE-1 cells. (D) Quantification of TRIM49 proteins presented in panel C. Relative expression of TRIM49 in relation to GAPDH.

**P* < 0.05, ns: no significance.


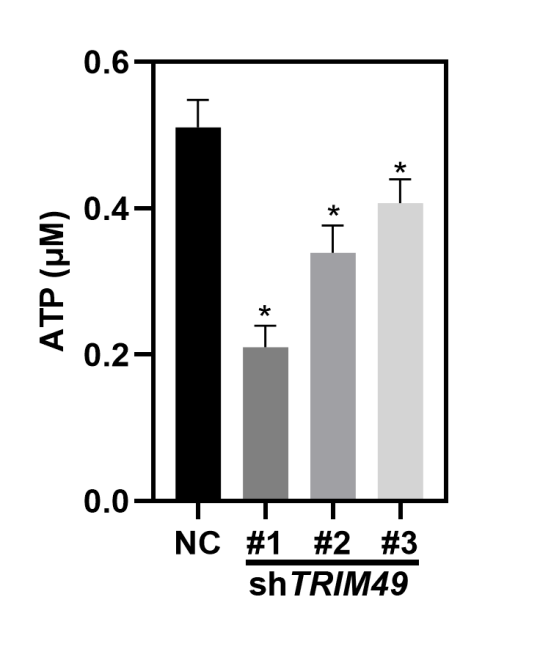


Fig. S5. ATP production of negative control (NC) and *TRIM49*-depleted hTERT RPE-1 cells.

**P* < 0.05. *N* = 3 for each group.


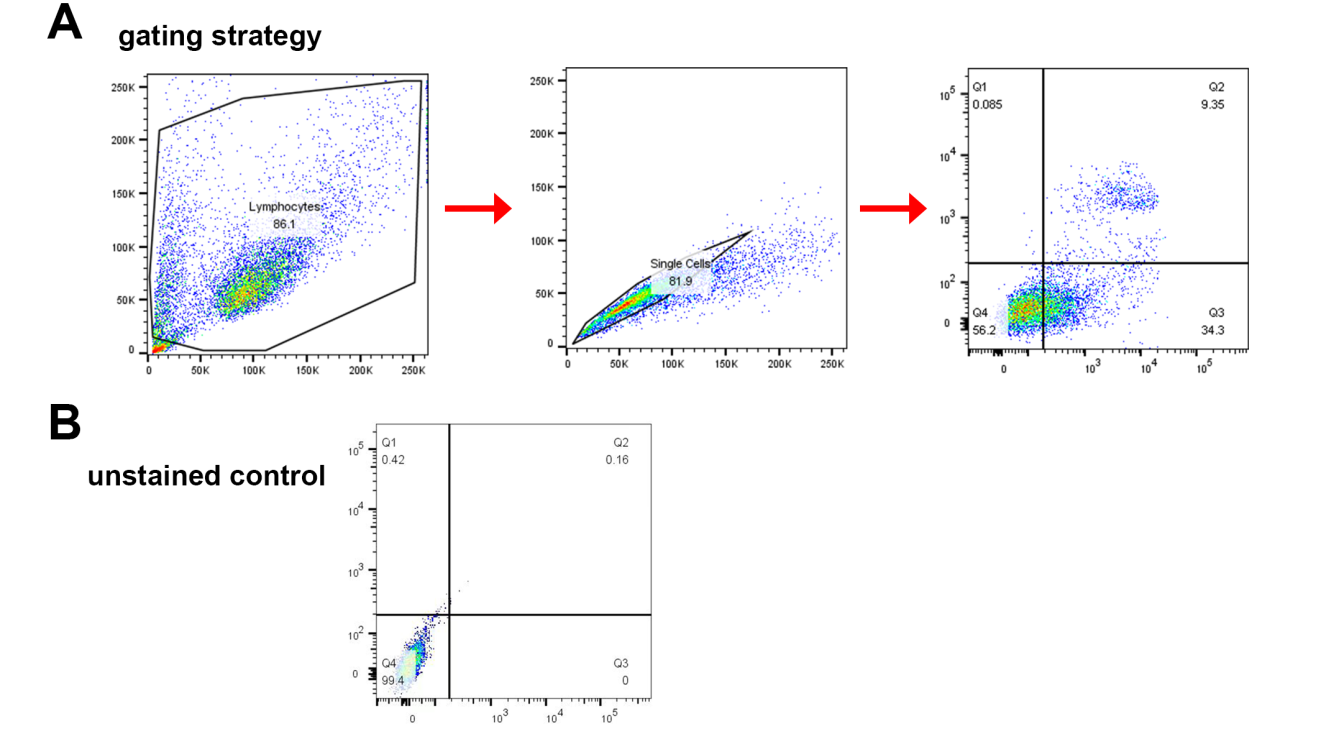


Fig. S6. Gating strategy and unstained control for flow cytometry of the apoptosis assay. (A) The gating strategy for flow cytometry. (B) The unstained control for flow cytometry.


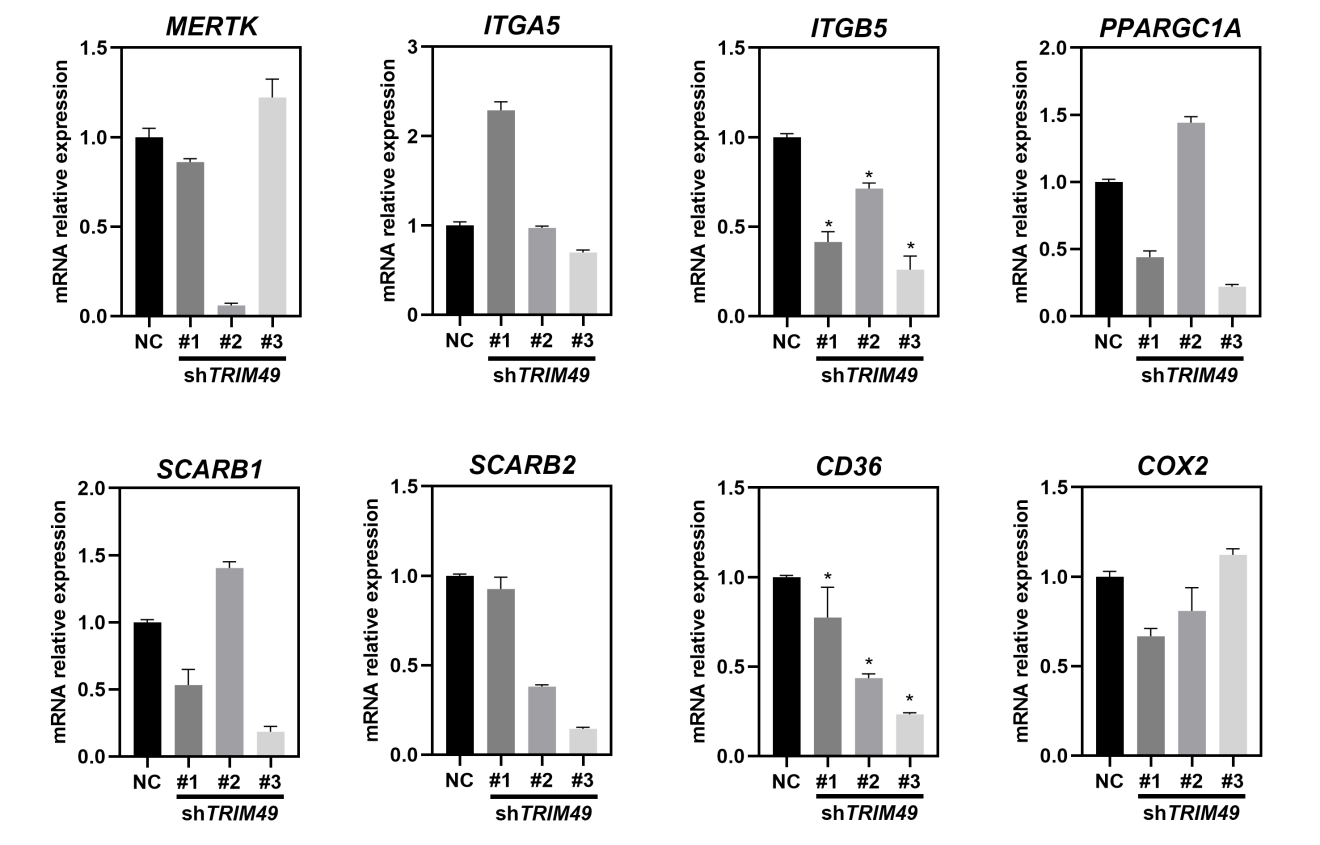


Fig. S7. *TRIM49* deficiency downregulates phagocytic receptors. Determination of the mRNA expression of numerous phagocytic receptors and associated molecules, including MERTK, αv integrin/ITGA5, β5 integrin/ITGB5, certain scavenger receptors (SCARB1, SCARB2, and CD36), PGC-1α/PPARGC1A and COX2 in normal control and *TRIM49*-depleted hTERT RPE-1 cells. Markedly decreased mRNA expression levels are seen for β5 integrin/ITGB5 and the scavenger receptor CD36 in *TRIM49*-depleted RPE cells. *N* = 3 for each group. **P* < 0.05.


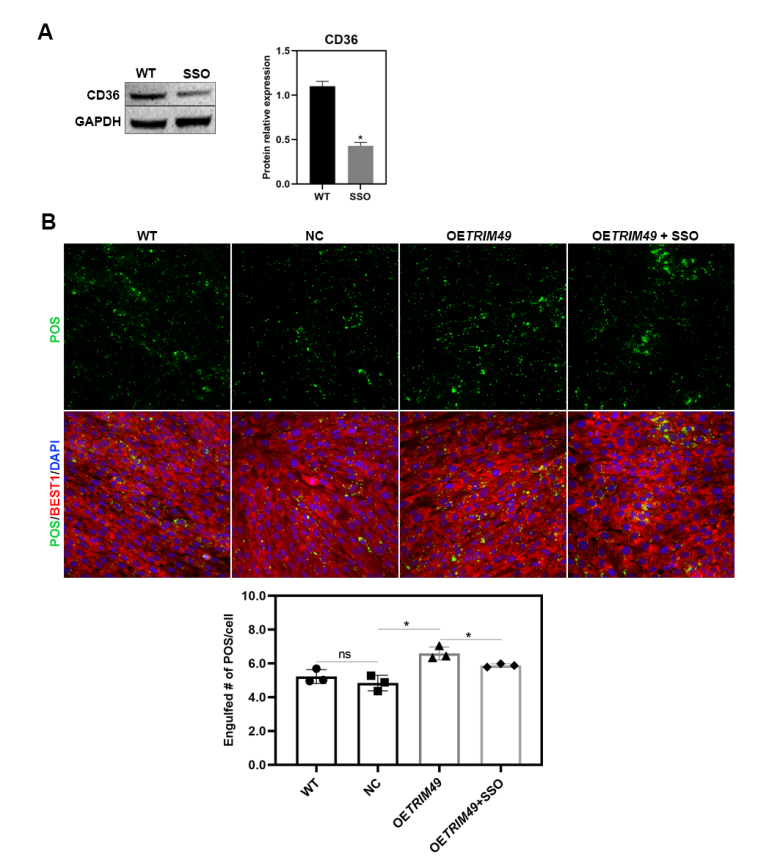


Fig. S8. Effect of CD36 inhibition on phagocytosis in *TRIM49* overexpressed RPE cells. (A) Sulfosuccinimidyl oleate (SSO) is a inhibitor of CD36. Left column: immunoblotting of CD36 in wild-type (WT) and SSO-treated RPE cells. Right column: quantification of CD36 proteins presented in left column. Relative expression of CD36 in relation to GAPDH. (B) Upper column: the hTERT RPE-1 cells were challenged with 10 FITC-labeled porcine photoreceptor outer segments (POS) per RPE cell for 6 hours. POS (green), BEST1 (red), DAPI (blue). Lower column: quantification of engulfed POS in wild-type (WT), normal control (NC), *TRIM49* overexpression (OE*TRIM49*) and SSO-treated *TRIM49* overexpression RPE cells. Cells were treated with 20 μM SSO. *N* = 3 for each group.


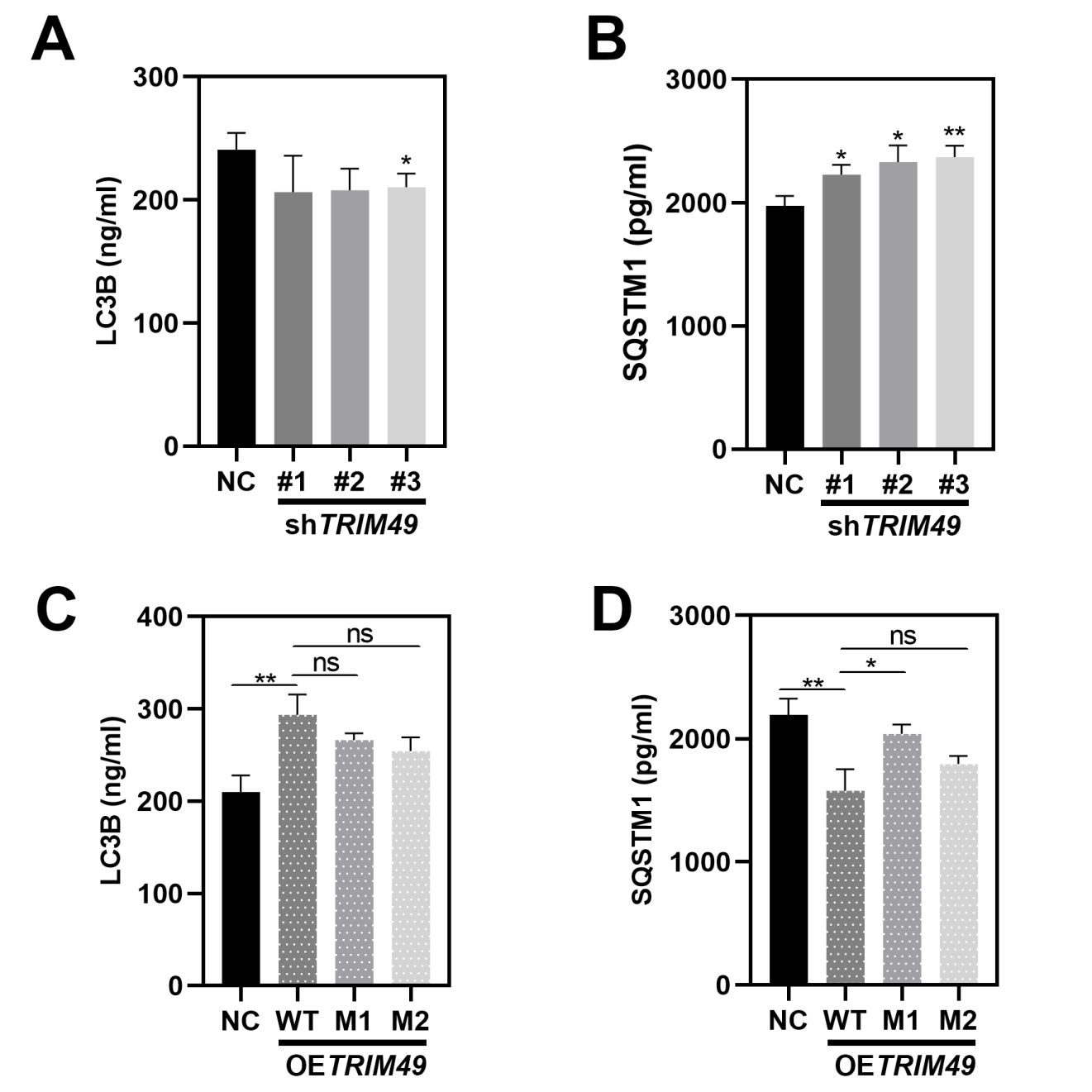


Fig. S9. Concentrations of LC3B and SQSTM1 detected by ELISA in hTERT RPE-1 cells with different experimental conditions. (A) ELISAs were performed to detect the concentrations of LC3B in lysates of NC and TRIM49-depleted RPE cells. (B) ELISAs were performed to detect the concentrations of SQSTM1 in lysates of NC and TRIM49-depleted RPE cells. (C) ELISAs were performed to detect the concentrations of LC3B in lysates of NC and TRIM49 overexpression RPE cells. (D) ELISAs were performed to detect the concentrations of SQSTM1 in lysates of NC and TRIM49 overexpression RPE cells. **P* < 0.05, ***P* < 0.01, ns: no significance versus NC group. *N* = 3 for each group. Error bars indicate standard deviation.


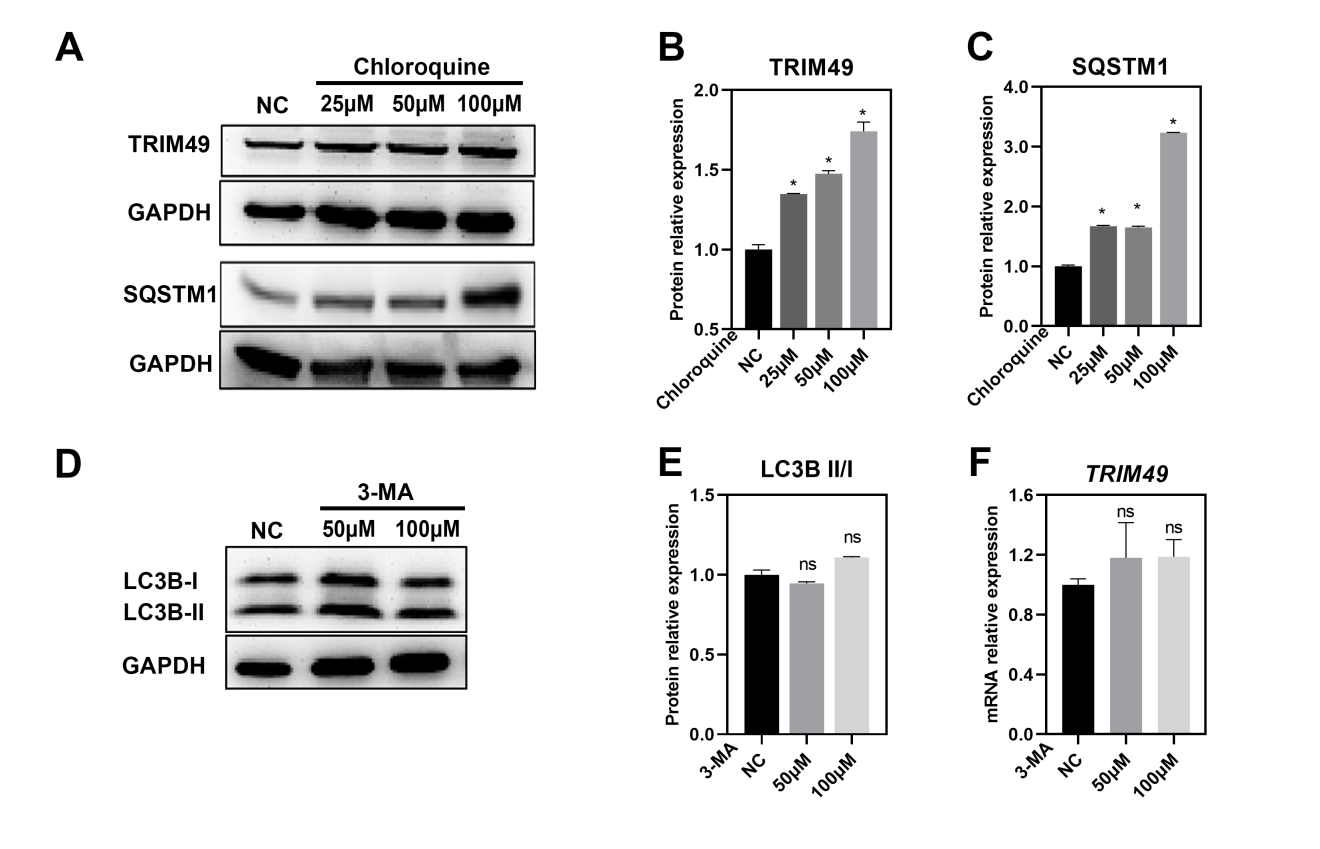


Fig. S10. Changes of TRIM49, LC3B, SQSTM1 or ULK1 expression in autophagy inhibitors treated human RPE cells. (A-C) Immunoblotting and quantification of the expression levels of TRIM49 as well as SQSTM1 in negative control (NC) and chloroquine-treated hTERT RPE-1 cells. (D-E) Immunoblotting and quantification of the expression levels of LC3BII/I in NC and 3-MA-treated hTERT RPE-1 cells. (F) Quantification of TRIM49 mRNA in NC and 3-MA-treated hTERT RPE-1 cells. ACTB was used as a normalizing gene.

**P* < 0.05, ns: no significance versus NC group. Error bars indicate standard deviation.


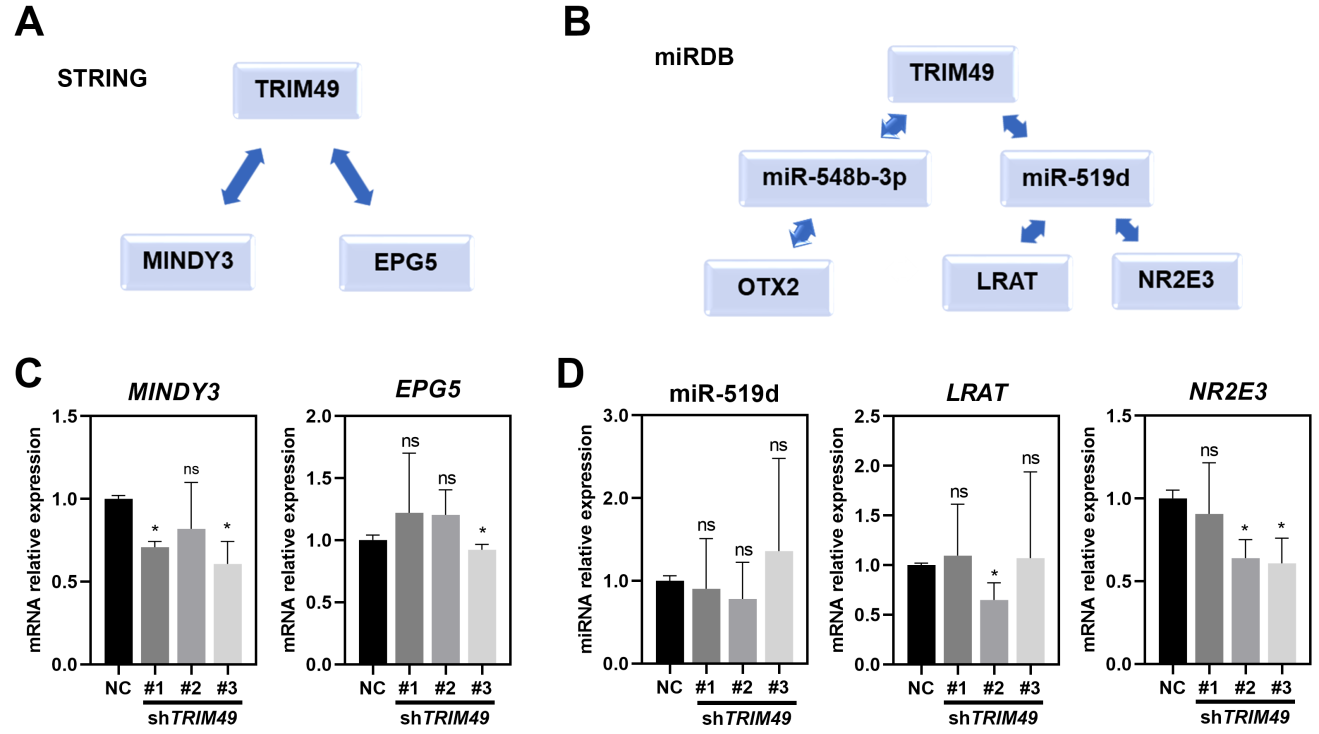


Fig. S11. Potential molecules interacting with *TRIM49* involved in retinal degeneration or visual cycle. (A) The STRING database predicted that FAM188A and EPG5 interacted with TRIM49. (B) The miRDB database predicted that the miRNA miR-548b-3p connected TRIM49 with OTX2 and that the miRNA miR-519d connected TRIM49 with LRAT and NR2E3. (C-D) Quantification of MINDY3, EPG5, miR-519d, LRAT and NR2E3 mRNA in NC and TRIM49-depleted hTERT RPE-1 cells. ACTB was used as a normalizing gene. *N* = 3 for each group. **P* < 0.05, ns: no significance versus NC group. Error bars indicate standard deviation.


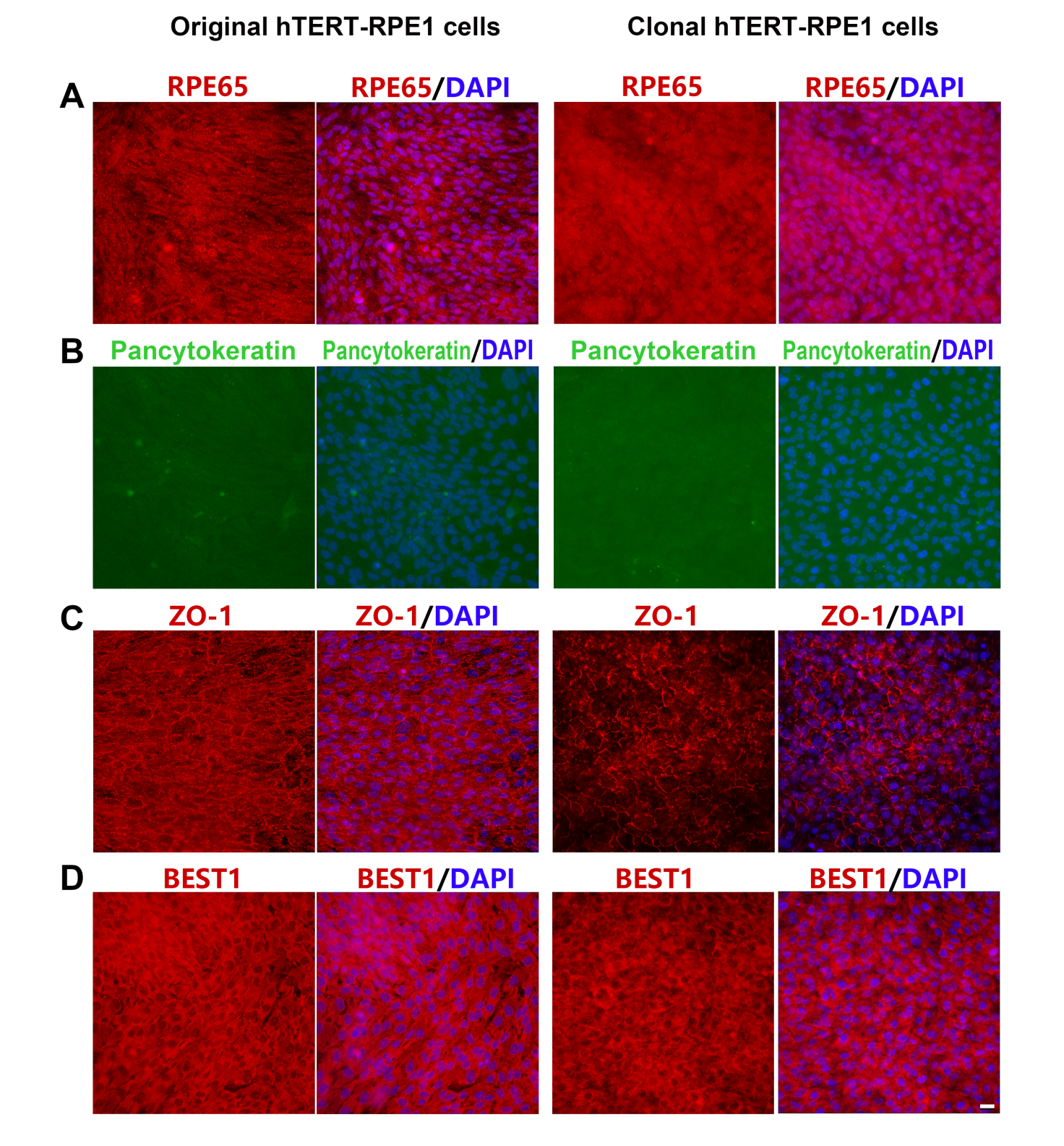


Fig. S12. Identification of original and clonal hTERT RPE-1 cells. Immunostainings for RPE-specific markers RPE65 (A, red), pancytokeratin (B, green), ZO-1 (C, red), and BEST1 (D, red) in both original (left panel) and clonal ones (right panel), confirming that the cells we used in this study are human RPE cells. The staining patterns in the clonal ones are similar to those in the original ones. DAPI is stained with blue. The scale bar represents 25 μm.


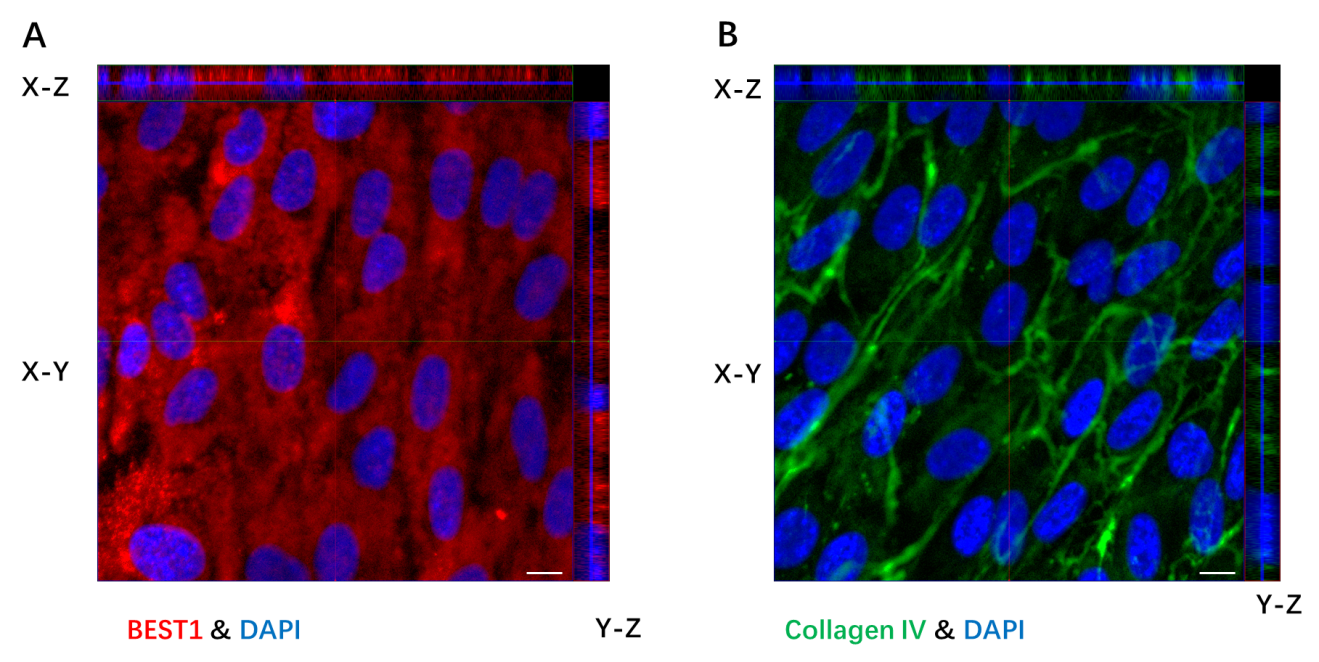


Fig. S13. Localization of basolateral markers in hTERT RPE-1 cells. Immunostainings for RPE basolateral markers BEST1 (A, red) and Collagen IV (B, green). For both markers, the localization in RPE includes from the basal to the apical, indicating that the RPE cells are not polarized. DAPI is stained with blue. Confocal X-Y, X-Z, and Y-Z scans were generated to show the localization of BEST1 and Collagen IV in the X, Y, and Z planes. The scale bar represents 10 μm.

**Supplementary tables**

Table S1. Rare variants in recessive known IRD genes detected in the two probands in single heterozygous state

| Proband ID | Chromosome position | Gene | Nucleotide and amino acid change | Reference transcript |
| --- | --- | --- | --- | --- |
| F1-II:1 | Chr16: 1561025 | IFT140 | c.4309G>A (p.Glu1437Lys) | NM_014714 |
| F2-IV:1 | chr11: 76873363 | MYO7A | c.1541G>T (p.Ser514Ile) | NM_000260 |
| F2-IV:1 | chr20: 2641159 | IDH3B | c.609C>G (p.Asp203Glu) | NM_006899 |

Table S2. Summary of bioinformatics analyses undertaken to predict the pathogenic nature of M1 (p.S395Y)

| Prediction tool | Predicted effect | Score |
| --- | --- | --- |
| PolyPhen-2  (http://genetics.bwh.harvard.edu/pph2/) | Probably damaging | 1.000  (the closer to 1, the more likely to be damaging) |
| PROVEAN  (http://provean.jcvi.org/index.php) | Deleterious | -4.08  (deleterious for a score equal to or below -2.5) |
| SIFT  (http://sift.jcvi.org/) | Damaging | 0.001  (damaging for a score below 0.05) |

Table S3. *TRIM49* primers for PCR/sequencing

| Variant | Forward primer (5’-3’) | Reverse primer (5’-3’) | Size(bp) |
| --- | --- | --- | --- |
| M1/M2 | GAAGATAGATGGAAAGGCGGG | GCTCACAGTCTTAGCCTCAC | 157 |

Table S4. Sequence of primers used for qPCR

| Name | Forward primer (5'-3') | Reverse primer (5'-3') |
| --- | --- | --- |
| *TRIM49* | CATCACTGGACTGAGGGACA | GTCTGAACACCCCATGCAAG |
| *ACTB*  (in 31 human tissues) | TGGCACCACACCTTCTACAA | CCAGAGGCGTACAGGGATAG |
| *ACTB*  (in hTERT-RPE1 cells) | TCGTGATGGACTCCGGTGAC | TCGTGGATGCCACAGGACTC |
| *MERTK* | CAGGAAGATGGGACCTCTCTGA | GGCTGAAGTCTTTCATGCACGC |
| *ITGA5* | GCCGATTCACATCGCTCTCAAC | GTCTTCTCCACAGTCCAGCAAG |
| *ITGB5* | GCCTTTCTGTGAGTGCGACAAC | CCGATGTAACCTGCATGGCACT |
| *SCARB1* | GGTCCAGAACATCAGCAGGATC | GCCACATTTGCCCAGAAGTTCC |
| *SCARB2* | GCCAATACGTCAGACAATGCCG | CTCATCTGCTTGGTAAAAGTGTGG |
| *CD36* | CAGGTCAACCTATTGGTCAAGCC | GCCTTCTCATCACCAATGGTCC |
| *CD81* | CTGCTTTGACCACCTCAGTGCT | TGGCAGCAATGCCGATGAGGTA |
| *PPARGC1A* | CCAAAGGATGCGCTCTCGTTCA | CGGTGTCTGTAGTGGCTTGACT |
| *PTK2* | GCCTTATGACGAAATGCTGGGC | CCTGTCTTCTGGACTCCATCCT |
| *COX2* | CGGTGAAACTCTGGCTAGACAG | GCAAACCGTAGATGCTCAGGGA |
| *TLR4* | CCCTGAGGCATTTAGGCAGCTA | AGGTAGAGAGGTGGCTTAGGCT |
| *TLR6* | ACTGACCTTCCTGGATGTGGCA | TGACCTCATCTTCTGGCAGCTC |
| *MINDY3* | AGGGCTCCAGTGGTCCAGAATC | GGAGTGTCATCTGTCTGTAGCATGG |
| *EPG5* | CAATGGCATGGTCTCGGCTCAC | CCTGTAGTCCTGGCTCCTTGGG |
| miR-548b-3p | GCGGCCTGAAAAGTAATTGTGGT | ATCCAGTGCAGGGTCCGAGG |
| *OTX2* | ATGCTGGCTCAACTTCCTACTTTGG | GACTGATTGAGATGGCTGGTGACTG |
| miR-519d | GATCATAACCTCCAAAGGGAAGCG | ATCCAGTGCAGGGTCCGAGG |
| *NR2E3* | GACCTCGGCTCGCCTACTCTTC | AGTTCACTCCACGCCTCTTCCAG |
| *LART* | GTCTCCAACAAGCGTCTCATCCTG | TAGGCGAAGTCCTCCACTGTGTC |

Table S5. Sequence of shRNA used for *TRIM49* knockdown

| **Name** | **Sequence (5'-3')** |
| --- | --- |
| shRNA1 | GATCGCGGAAAGAGAAGAATCAGAATCTCGAGATTCTGATTCTTCTCTTTCCGTTTTTGAATT |
| shRNA2 | GATCGACCAGCCGAGTAGGATTATTCCTCGAGGAATAATCCTACTCGGCTGGTTTTTTGAATT |
| shRNA3 | GATCGACTTTCACCTCGGGCAAATATCTCGAGATATTTGCCCGAGGTGAAAGTTTTTTGAATT |

Table S6. List of primary antibodies used for western blotting

| Antibody | dilution | Supplier |
| --- | --- | --- |
| TRIM49 | 1:800 | BIOSS, bs-16742R |
| LC3B | 1:1000 | Sigma, L7543 |
| SQSTM1 | 1:1000 | Boster Biological Technology, PB0458 |
| ULK1 | 1:800 | HUABIO, H661367011 |
| OTX2 | 1:800 | Proteintech, 13497-1-AP |
| CD36 | 1:100 | HUABIO, H661707014 |
| β5 integrin/ITGB5 | 1:100 | Cell Signaling Technology, 4711S |
| GAPDH | 1:5000 | Bioworld, AP0063 |
| β-tubulin/TUBB | 1:5000 | Bioworld, MB0009 |
